# Revealing the therapeutic potential of bioactive exopolysaccharide (EPSS10) derived from *Streptomyces* sp. MAE

**DOI:** 10.1186/s12866-025-04462-x

**Published:** 2025-11-15

**Authors:** Esraa S. Refat, Attia A. Attia, Mohamed E. El Awady, Mohamed Ali, Ahmed A. Hamed, Mohamed A. Nasr-Eldin

**Affiliations:** 1 Botany and Microbiology Department, Faculty of Science, Benha University, Benha, 13511 Egypt; 2 Microbial Biotechnology Department, National Research Centre, El- Buhouth St. 33, Dokki, Cairo, 12622 Egypt; 3 Biochemistry Department, Faculty of Science, Zagazig University, Zagazig, 44519,, Egypt; 4 Microbial Chemistry Department, National Research Centre, El-Buhouth St. 33, Dokki, Cairo, 12622 Egypt

**Keywords:** Streptomyces, Exopolysaccharide, Antioxidant, Antimicrobial, Anti-inflammatory, Antitumor, Real-time PCR, Antiviral

## Abstract

Currently, there is an increasing interest in microbial exopolysaccharides (EPSs) due to their diverse functionalities, diversity in chemical structure and source, and enormous potential for use in different medical applications. A novel marine *Streptomyces* sp. MAE (OR354383) was isolated and identified that generated a significant quantity of exopolysaccharide (EPS) at 7.6 g/L. Bioactive exopolysaccharide (EPSS10) was extracted and characterized from *Streptomyces* sp. MAE culture, and the molar ratio was determined via HPLC. Several methodologies were used to determine their antioxidant, antimicrobial, anti-inflammatory, anticancer, and antiviral activities. The anticancer efficacy of EPSS10 was evaluated using several cell lines (HepG2, CaCo2, MCF7, PC3, A549 and PANC1). Using Real time (RT)-PCR, we analyzed the expression levels of *Bax*,* Bcl2*,* p53*,* Cytochrome c*, and *Caspase 9* in MCF7 cells. EPSS10 displays a grayish-white coloration, possesses an amorphous structure, and is devoid of odor. The composition of EPSS10 includes uronic acid (19.55%), sulfate (25.03%), and N-acetylglucosamine (10.63%). The in vivo toxicity results proved the safety of EPSS10 for biological parameters in rat models. EPSS10 demonstrates significant antibacterial efficacy. The maximum antioxidant was 89.21 ± 0.3% at 500 µg/ml; anti-inflammatory activity as cyclooxygenase (COX-1) inhibitory showed that the inhibition percentage was 81.9 ± 0.5%, and cyclooxygenase (COX-2) inhibitory provided 78.0 ± 1.2%. The fraction EPSS10 had a strong cytotoxic effect on different cancerous cells by increasing their concentrations. EPSS10 treatment increased *Bax*,* Caspase 9*,* Cytochrome c*, and *p53* gene expression. The expression of the *Bcl2* gene, however, was reduced. The antiviral efficacy of EPSS10 was determined to be 57.29 ± 1.78% at the maximum non-cytotoxic dose, whereas the concentration required to achieve a 50% reduction in the pathogenic effects of the Hepatitis A Virus (HAV) was 838.50 ± 34.62 µg/ml. The exopolysaccharide (EPSS10) of *Streptomyces* sp. MAE has important therapeutic potential due to its significant biological functions, safe use, structural features, and intrinsic qualities.

**Supplementary Information:**

The online version contains supplementary material available at 10.1186/s12866-025-04462-x.

## Introduction

Marine microorganisms have been known to produce a wide range of beneficial compounds, such as biopolymers and secondary metabolites, and they produce extracellular polysaccharides and other compounds for protection against harsh environmental conditions, such as hypersalinity, pH, and predation. Streptomycetes are Gram-positive bacteria and have a complicated life cycle. They generate a variety of secondary metabolites with a range of structures and purposes, including herbicides, antimicrobials, anticancer, and immunosuppressive medications. As a result, *Streptomyces* are thought to be the most well-known bacteria in the fermentation process used to produce active pharmaceutical ingredients [1]. Exopolysaccharides (EPSs) are extracellular carbohydrate polymers that are synthesized by different microorganisms [2, 3], they can also be freely released into the culture media, such as slime EPS, or they can be a putative capsular polysaccharide (CPS).

Microbial EPSs offer a wide range of functions, including surface adhesion, stabilizing enzymes, storing nutrients, protecting microorganisms from phagocytosis, forming biofilms, and protecting parasitic organisms from desiccation and predation [4, 5]. Moreover, EPSs have demonstrated a wide range of industrialized applications, including emulsification, thickness, absorption, film creation, gel formation, anticancer treatment, etc. EPSs have strong biological activities that regulate immune function, cell division, and differentiation, and have antiviral, antitumor, and antioxidant properties that prevent atherosclerosis and inflammation [6–9].

The antiviral effects of microbial EPSs can be either systemic or local. When acting in the local mode, the EPSs engage in direct contact with the viruses or the host cell's receptors. Thus, microbial EPSs can either suppress viral replication enzymes or boost innate and adaptive immunity in systemic mode actions by blocking viral adsorption [10].

Hepatitis A virus (HAV) infects hepatocytes and causes a range of clinical symptoms, from asymptomatic infection to acute, fulminant hepatitis. HAV spreads through the oral-fecal pathway. After an incubation period of 15 to 40 days, symptoms—which are frequently gastrointestinal in nature—appear suddenly [11].

Cancer poses a major challenge in terms of social, public health, and economic factors, responsible for approximately one in six fatalities (16.8%) and one in four deaths (22.8%) attributed to noncommunicable diseases (NCDs) worldwide. NCDs account for three out of ten premature deaths globally (30.3% among individuals aged 30 to 69), and in 177 of the 183 countries studied, cancer is listed as one of the top three leading causes of death within this age demographic [5, 12].

Microbial EPSs have demonstrated in vitro antiproliferative characteristics against a range of cancers, such as cervical, breast, pancreatic, colon, and leukemia [13]. The anticancer efficacy of microbial EPSs is determined through their molecular structure, molecular weight, uronic acid and sulfate groups, and β-type glycosidic linkages [14]. According to Koller et al. [15], microbial EPSs most likely carry out their anticancer actions by acting as antioxidants, binding to genotoxic carcinogens, inducing apoptosis, and enhancing immunity. Additionally, microbial EPSs can exert their anticancer effects by inducing apoptosis. Both intrinsic and extrinsic caspase-dependent pathways can cause apoptosis. *Caspases 3*,* 9*,* BCl2*, and *Bax* are expressed in the intrinsic pathway, while *caspases* 8 and 10 are expressed in the extrinsic pathway. The activation of *caspase-3* signifies that malignant cells have experienced chromatin condensation, nuclear fragmentation, and cell shrinkage, while leaving adjacent healthy cells and tissues unaffected [16].

Moreover, microbial EPS can stimulate cell-mediated immune responses, such as the phagocytic ability of mononuclear cells, the proliferation of T-lymphocytes, and the tumoricidal activity of natural killer cells, to produce their anticancer effects. According to Wu et al. [17], *L. lactis* subsp. lactis EPS has been shown to increase intracellular calcium levels and produce inflammatory cytokines by inducing MCF7 cell apoptosis, nuclear condensation, and cell shrinkage.

The present work is carried out to isolate and identify exopolysaccharide-producing *Streptomyces* sp. additionally, it investigates pharmaceutical and medicinal uses related to its activity as an antiviral, anticancer, antibacterial, anti-inflammatory, and antioxidant.

## Materials and methods

### Collection of samples and isolation of streptomycetes

Samples were obtained from the sediment at a depth of 1 cm along the shoreline of the Red Sea in Hurghada, Egypt (27°18̀22.02̏ N, 33°43 `44.8̏ E). The sample was subjected to serial dilution according to Hayakawa and Nonomura [18]. It was then overlaid on starch nitrate agar medium, which was prepared by dissolving the medium in 750 ml of seawater and adjusting the volume to 1 L with 250 ml of distilled water, maintaining a pH of 7 [19]. Subsequently, the Streptomycetes isolates were selected and collected based on their morphological characteristics.

## Identification of bacterium that produces EPS

The isolate S10 that produces EPS was selected and identified through its morphological, physiological, and biochemical characteristics; additionally, the confirmation was performed using the 16 S ribosomal RNA gene method (16 S rRNA), employing the primers F-(5'-GAGTTTGATCCTGGCTCAG-3') and R-(5'-GGTTACCTTGTTACGACTT-3') as described by Gardes and Bruns [20]. Following sequencing, the obtained 16 S rRNA sequence was submitted to the GenBank database. Subsequently, it was compared with other 16 S rRNA sequences accessible through the National Center for Biotechnology Information (https://www.ncbi.nlm.nih.gov/) using the BLAST tool. The sequences of bacterial strains most comparable to the 16 S rRNA gene of our isolate were selected and aligned to construct an appropriate phylogenetic tree.

## Production, extraction, and purification of exopolysaccharides (EPS)

A loop containing strain S10 was introduced into a 250 ml conical flask containing 50 ml of broth medium (g/l). The medium was prepared by dissolving 10.0 g of glucose, 5.0 g of tryptone, 5.0 g of yeast extract, 3.0 g of K_2_HPO_4_, 3.0 g of NaCl, 1.0 g of KH_2_PO_4_, 0.5 g of MgSO_4_.7H_2_O, and 0.5 g of CaCO_3_ in 750 ml of seawater. The solution was then brought to a final volume of 1 L by adding 250 ml of distilled water, with the pH adjusted to 7 [21]. The inoculation medium was incubated on an incubator shaker at 120 rpm for five days at a temperature of 28 °C. Following this, the supernatant from the cell-free culture was centrifuged at 5000 rpm for 20 min. The supernatant was then combined with 10% trichloroacetic acid (TCA) and allowed to incubate overnight at 4 °C under static conditions. Afterward, it was centrifuged again at 5000 rpm for 20 min to eliminate proteins, and the pH of the supernatant was adjusted to 7 using an NaOH solution [22]. The EPS solution was subsequently diluted fourfold with cold alcohol and stored at 4 °C overnight. The precipitated EPS was centrifuged at 5000 rpm for 20 min to separate EPS, then resuspended in deionized water and washed twice. They were subsequently re-precipitated using four volumes of cold 100% ethanol and dehydrated at 50 °C. For the purpose of fractionation and initial fraction identification, absolute alcohol was added sequentially in volumes of 1, 2, 3, and 4, leading to the collection of the corresponding precipitated EPS. The primary fraction derived from three liters underwent dialysis against distilled water for 72 h, followed by two acetone rinses, ether dehydration, and drying at 40 °C [23]. Following concentration, the active ingredients were combined and dialyzed with deionized water. Fractions from the previous step were concentrated and dialyzed against distilled water, then adsorbed on a DEAE-cellulose column (70 × 1.5 cm) equilibrated with distilled water. Non-absorbed exopolysaccharides were washed with distilled water, and the absorbed exopolysaccharides were eluted with a stepwise gradient of NaCl (0.2–3.0.2.0 M). Fractions of 5.0 ml/5 min were collected [24]. Then, it was analyzed by the phenol-sulfuric acid method [25]. The active fractions were pooled together, dialyzed against deionized water, and precipitated with ethanol after concentration. EPSS10 fraction powder was employed for characterization and the examination of biological activity.

## Chemical analysis of EPSS10

EPSS10 underwent comprehensive acid hydrolysis using 88% formic acid at 100 °C for 5 h. The concentration of sulfate was determined using the turbidity technique with Barium Chloride-Tween 80 reagent, as outlined by Dodgson and Price [26]. The uronic acid concentration was quantified using spectrophotometry at a wavelength of 525 nm with a Shimadzu 2401PC 750 Lambda Double Beam UV–Vis spectrophotometer, in accordance with the m-hydroxybiphenyl colorimetric technique [27]. For the investigation of monosaccharide structure and the calculation of molar ratios, high-performance liquid chromatography (HPLC, Agilate Pack, series 1, 200) was utilized, featuring an Aminex carbohydrate HP-87 C column (300 × 7.8 mm) and employing deionized water as the mobile phase at a flow rate of 0.5 ml/min [28]. A 2 mg sample of dried EPSS10 was combined with 200 mg of KBr powder and subsequently ground into a pellet measuring 1 mm for Fourier-transform infrared (FTIR) spectral analysis. It was conducted over a wavelength range of 400–4000 cm^−1^ by the FTIR-UNIT Bruker Vector 22 Spectrophotometer [29].

## Assessment of systemic acute toxicity properties of EPSS10

### Animal

A cohort of ten male Sprague-Dawley rats, each with a weight ranging from 100 to 150 g, was provided by the Animal House at the National Research Centre in Cairo, Egypt. These rats were then housed in polycarbonate enclosures, each designed to hold a maximum of four rats. They were kept in a controlled setting featuring a 12-hour light-dark cycle, with the temperature maintained at 24 ± 2 °C through an air conditioning system. All procedures were carried out in accordance with the appropriate norms and guidelines; the lethal toxicity (LD_50_) of EPSS10 was assessed in male rats using the Meier method; a systematic sequence of doses, beginning at 500 mg and increasing in concentration, was administered; all injections were performed via the intraperitoneal route; and the moisture content was kept between 50% and 70%. The handling and all experiments complied with the ARRIVE criteria and were approved in accordance with guidelines approved by the IACUC (Institutional Animal Care and Use Committee) of Benha University, Egypt No. [Bufs-REC-2024-98Bot]. Twenty-four hours following injection, the treated subjects' mortality and survival rates were tracked. Ten white male rats were used in this study to evaluate the effects of EPSS10 on the liver, brain, kidneys, and heart, among other important organs. Oral doses of EPSS10 were administered to the rats based on their body weight. For a total of 14 days, the administration was conducted every day. At the conclusion of the study session, on Day 14, all rats were euthanized [30].

## Preparing a blood sample and biochemical parameters

At the end of the study period, the rats were administered anesthesia with ketamine and xylazine at a dosage of 80–100 mg/kg of ketamine and 10–12.5 mg/kg of xylazine. Then, all experimental rats were euthanized by carotid exsanguination just after loss of consciousness. Blood samples were obtained from the retroorbital venous plexus using capillary tubes that were treated with heparin. The blood samples were subjected to centrifugation at a speed of 3000 rpm for a duration of 10 min. The sera were kept at a temperature of −20 °C until they were analyzed to determine biochemical characteristics. An automated biochemistry analyzer was used to ascertain the parameters as the levels of lactate dehydrogenase (LDH), blood urea nitrogen (BUN), aspartate aminotransferase (AST), and alanine aminotransferase (ALT) in serum. Numerous parameters were measured as part of the hematological parameter analysis process, such as white blood cells (WBC), reticulocytes (RET), platelets (PLT), mean corpuscular hemoglobin concentration (MCHC), mean corpuscular hemoglobin (MCH), hematocrit (HCT), mean cell volume (MCV), and mean corpuscular hemoglobin (MCH).

## Histological evaluation

10% of neutral buffered formalin was used to preserve liver, heart, kidney, and brain specimens obtained from every test. Before being incorporated in paraffin wax, the samples underwent a series of ethanol solutions to dehydrate them after fixation. Thereafter, xylene was used to clean the specimens. A microtome was utilized to generate sections measuring 5 μm in thickness. Following staining with hematoxylin and eosin (H and E), these sections were examined under a light microscope.

### Assessment of EPSS10 antimicrobial activity

To assess the antibacterial efficacy of EPSS10, experiments were conducted utilizing 96-well polystyrene flat plates. A total of ten microliters of sample extracts (resulting in a final concentration of 500 µg/ml) were combined with eighty microliters of lysogeny broth (LB broth). This mixture was then supplemented with ten microliters of isolated bacteria in suspension (during the log phase) and incubated overnight at 37 °C. *Bacillus subtilis* ATCC-6633 and *Staphylococcus aureus* NRRLB-767 exemplify gram-positive bacteria. *Escherichia coli* ATCC-25,922 and *Pseudomonas aeruginosa* ATCC-10,145 exemplify gram-negative bacteria, while yeast (*Candida albicans* ATCC-10231) and fungus (*Aspergillus niger* NRRLA-326) functioned as test organisms. Further, the plates were incubated at 37 °C overnight for bacteria and for 28 °C fungi. The absorbance was determined as the mean standard deviation (SD) using a Spectrostar Nano Microplate Reader (BMG LABTECH GmbH, Allmendgrun, Germany) after approximately 20 h at OD600 [31].

### Assessment of EPSS10 antioxidant activity

#### DPPH free radical scavenging activity

As the Brand Williams, et al. [32],, a mixture was prepared by combining 200 µL of a 0.1 mM DPPH and ethanol solution with 100 µL of an EPSS10 and positive control (Ascorbic acid) solution, which had varying concentrations (100 to 500 µg/mL). At 37 °C, the mixture underwent incubation for 30 min. At a wavelength of 517 nm, the test mixture's absorbance was measured. The reference material was ascorbic acid, while ethanol was used as a blank control. The scavenging activity of the DPPH radical was assessed as follows:

1 $$\begin{aligned}&DPPH\;Scavening\;activity\;\left(\%\right)\;=\\&\;\left[\left(A\;control\;-A\;sample\right)\div A\;control\right]\\&\times100\end{aligned}$$

### ABTS scavenging activity

According to the technique outlined by [33], the ABTS radical cation scavenging action of EPSS10 and the positive control (Ascorbic acid) were assessed at distinct doses ranging from 100 to 500 µg/ml. The following calculation was carried out to determine the ABTS radical cation scavenging activity.

2 $$\begin{aligned}&ABTS\;Scavening\;activity\;\left(\%\right)=\\&\left[\left(A\;control\;-\;A\;sample\right)\div A\;control\right]\\&\times100\end{aligned}$$

### Ferrous reducing antioxidant capacity assay

By using the Oyaizu technique [34], The ferric reducing antioxidant capability (FRAC) of the EPSS10 and positive control (Ascorbic acid) were assessed. The reaction mixture's enhanced reducing power is indicated by its increased absorbance. At each concentration, the experiment was carried out three times.

### Lipid peroxidation Inhibition assay

The lipid peroxidation inhibition assay was conducted for EPSS10 and positive control (Ascorbic acid) following the procedure outlined by Gulcin et al. [35]. The following formula was used to calculate the samples' percentage of lipid peroxidation inhibition:

3 $$\begin{aligned}&Liquid\;peroxidation\;inhibition\;\left(\%\right)\;=\\&\;\left[\left(A\;control\;-\;A\;sample\right)\div A\;control\right]\\&\times100\end{aligned}$$

### Superoxide radical scavenging activity

In accordance with the established methodology [36], The superoxide anion scavenging activity was evaluated for EPSS10 and positive control (Ascorbic acid). Superoxide radicals were produced through the oxidation of NADH in a PMS-NADH system and quantified by the reduction of NBT. The proportion of superoxide radical scavenging was determined by the formula:

4 $$\begin{aligned}&Superoxide\;Scavening\;activity\;\left(\%\right)\;=\\&\;\left[\left(A\;control\;-\;A\;sample\right)\div\;A\;control\right]\\&\times100\end{aligned}$$

### Nitric oxide scavenging activity

According to the procedure outlined by Adithya et al., [37], the nitric oxide scavenging test was carried out for EPSS10 and the positive control (Ascorbic acid). The reaction mixture comprised 0.5 mL of PBS and 2 mL of sodium nitroprusside at a concentration of 10 mM (pH 7.4). The reaction mixture was administered at 0.5 mL at a concentration of 20–100 g/mL, subsequently agitated, and incubated for 2.5 h at ambient temperature. A 0.5 mL aliquot of the mixture was extracted, combined with 1 mL of 0.33% sulfanilic acid in a separate test tube, and allowed to stand for 5 min at ambient temperature. Subsequently, 1 mL of 0.1% naphthalene diamine chloride is introduced, and the mixture is incubated for 30 min at ambient temperature. The absorbance was quantified at 540 nm.

### Evaluation of anti-inflammatory activity by in vitro suppression of cyclooxygenase enzymes (COX-1 & COX-2)

The inhibitory activity of Exopolysaccharide (EPSS10) with respect to assessing cyclooxygenase (COX-1) and cyclooxygenase (COX-2); the COX Colorimetric Inhibitor Screening Assay Kit (Cayman, Ann Arbor, MI, USA) was utilized with sample concentrations (50–500 µg/ml), in accordance with the manufacturer's instructions and the method of Lee et al., [38] with minor modifications. The percentage of COX inhibition was assessed as follows:

5 $$\begin{aligned}&COX\;inhibition\;activity\;=\;\\&\left(1-As\div Ac\right)\times100\end{aligned}$$

Whereas, *As* is the absorbance of the test drug, and *Ac* is the absorbance of the control.

### Invitro assessment of antitumor activity

Cell lines for hepatocellular carcinoma (HepG2), colorectal adenocarcinoma (CaCo2), breast adenocarcinoma (MCF7), lung adenocarcinoma (A549), prostate adenocarcinoma (PC3), and pancreatic adenocarcinoma (PANC1) were obtained from the Tissue Culture Unit, VACSERA, Egypt, for the purpose of evaluating their cytotoxicity and viability. 10% fetal bovine serum (FBS) from Life Science Production Group LPS, UK, 1% glutamine, and 1% penicillin/streptomycin from the Lonzo group, Verviers, Belgium, were added to RPMI 1640 media in order to nourish the cells. The cells were kept in a humidified incubator with 5% CO2 at 37 °C. The Methyl Thiazolyl Tetrazolium (MTT) viability test was used to assess the cytotoxicity of EPSS10 [39]. Every test was run three times, and the average of the results was calculated. The following formula was used to determine cell viability:

6 $$\begin{aligned}&Cell\;viability\;\left(\%\right)=\;\\&\left[1-(O.D.\;test\;\div\;O.D.\;control\right]\\&\times100\end{aligned}$$

### Cell line treatment

After being treated with EPSS10 at a concentration of 78.12 µg/mL for 24 h, the MCF7 cell line was used for real-time polymerase chain reaction (RT-PCR).

### RNA extraction and real-time PCR analysis

Total RNA was extracted from both EPSS10-treated and untreated cells utilizing the Qiqamp mini kit (Qiagen; USA) in accordance with the manufacturer's guidelines. Following the RNA extraction, its quality and quantity were evaluated through UV spectrophotometry with a Nanodrop spectrophotometer (Thermo Scientific). Real-time RT-PCR was conducted as described by Piran et al., [40], and gene expression levels were evaluated using specific primers (supplementary materials Table 1). The gene GAPDH was considered housekeeping. Using the comparative CT (2^−ΔΔCT^) methodology, the relative expression of each gene under study was determined.

## Assessment of cytotoxicity and antiviral activity

### Virus and cell line propagation and cytotoxicity Estimation

The cytopathogenic HAV was cultured and evaluated in confluent Vero cells, as described by Randazzo et al. [41]. The infectious virus quantification was carried out by determining the 50% tissue culture infectious dose (TCID50), utilizing eight wells for each dilution and 20 µL of inoculum per well, in accordance with the Spearman-Karber method (Pinto et al., 1994). Vero cells, obtained from the kidneys of the African green monkey, were acquired from the American Type Culture Collection (ATCC, Manassas, VA, USA). These cells were cultured in Dulbecco's modified Eagle's medium (DMEM) enhanced with 10% heat-inactivated fetal bovine serum (FBS), 1% L-glutamine, HEPES buffer, and 50 µg/mL gentamicin. They were kept at 37 °C in a moistened environment containing 5% CO2 and were sub cultured biweekly [42]. The Vero cell lines utilized for the cytotoxicity measure were plated in 96-well plates at a concentration of 2 × 10^5 cells per ml, utilizing 100 µL of development medium. New medium containing varying concentrations of EPSS10 was presented to the blended cell monolayers in flat-bottom microtiter plates (Falcon, Jersey, NJ, USA). The microtiter plates were put in a humidified incubator set at 37 °C with 5% CO_2_ and permitted to incubate for a term of 48 h. The supernatant was disposed of, and the cells were washed twice. An MTT test was utilized to survey cell viability [39].

### Antiviral activity

Antiviral screening was conducted employing a cytopathic effect inhibition test for the HAV at the Regional Center for Mycology and Biotechnology (RCMB) at Al-Azhar University, Cairo, Egypt. This assay successfully exhibited the targeted inhibition of a biological function, particularly the cytopathic effect in vulnerable mammalian cells, which was evaluated through the MTT technique [43]. The rate of viral inhibition was determined using the following method:

7 $$\begin{aligned}&The\;rate\;of\;viral\;inhibition\;=\\&\;\left[\left(A-B\right)\div\left(C-B\right)\right]\times100\end{aligned}$$

A, B, and C represent the absorbance values of the compounds being studied in virus-infected cells, the absorbance of the viral control, and the absorbance of the cell control, respectively.

### Statistical analysis

All assays were estimated in triplicate, and the results were presented as means ± standard deviation (SD). Statistical analysis was performed using variance analysis via GraphPad Prism version 10.5.0, with significance determined at a *p-value*.

## Results

### Isolation and identification of marine streptomycetes

**Fig. 1:**
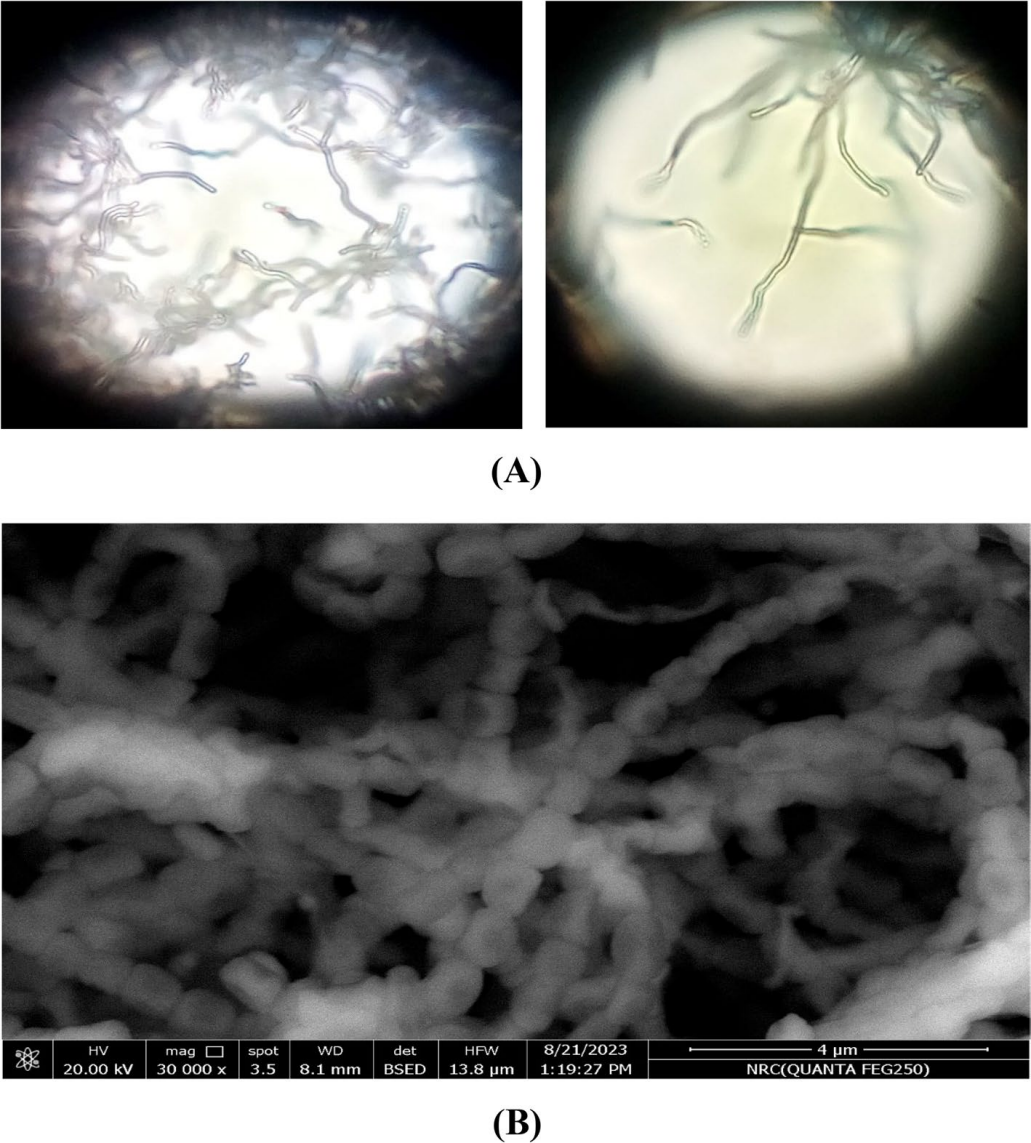
Photomicrograph of S10 showing straight sporephores hyphae (**A**) & scanning electron microscope (SEM) photomicrograph of S10 showing smooth spore surface (**B**)

**Fig. 2:**
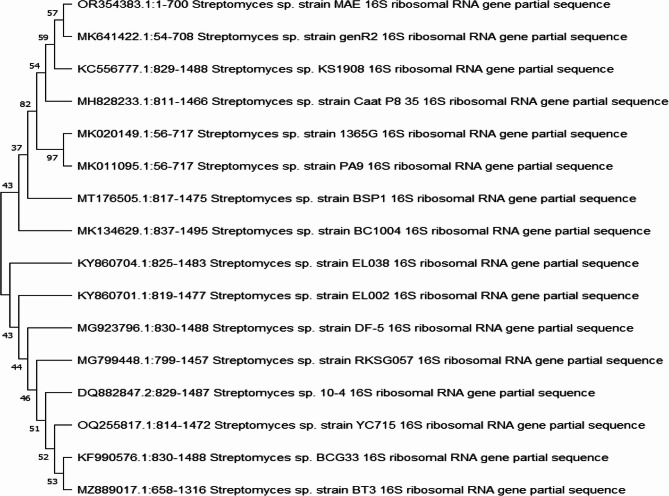
Phylogenetic tree of the partial sequence of 16S rRNA of the isolate *Streptomyces* sp. MAE isolate (S10) respects to closely related sequences available in GenBank databases

A total of twelve isolates were obtained from the sediment of the Red Sea in Hurghada, Egypt. Among these twelve isolates (S1 to S12), only one isolate, S10, demonstrated a high polysaccharide production of 7.6 g/L. Isolate S10 exhibited straight spore chain morphology with a smooth surface ornamentation on the spores (Fig. 1). The color of the spore mass was gray, and the substrate mycelium pigmentation was pale gray. It can't produce diffusible pigments or melanoid pigments on tyrosine agar. Elastin and arbutin were degraded actively, reducing nitrate and producing hydrogen sulfide. Utilization of sugars has been performed by using D-glucose as a positive control. It can utilize D-mannitol, D-xylose, galactose, and arabinose **(**Table 1**)**. A phylogenetic analysis of the 16 S rRNA gene sequence of strain S10 was conducted, comparing it to reference sequences available in the GenBank and EMBL databases, which were retrieved from the National Centre for Biotechnology Information using a BLAST search (http://ncbi.nlm.nih.gov/BLAST/), as illustrated in Fig. 2. They were recognized as *Streptomyces* sp. MAE under accession number OR354383.

**Table 1:** Morphological, physiological and biochemical characteristics of S10 isolate

Morphological and cultural characteristics	
Spore chain morphology	Straight
Spore surface ornamentation	Smooth
Color of spore mass	Gray
Pigmentation of substrate mycelium	Pale Gray
Diffusible pigment	- ve

**Physiological and biochemical characteristics**	
Melanin pigment Production	- ve
Nitrate reduction	+ ve
H_2_S production	+ ve

**Utilization of sugars**	
D-fructose	- ve
Sucrose	- ve
Rhamnose	- ve
D-mannitol	+ ve
D-xylose	+ ve
Raffinose	- ve
I-inositol	- ve
Galactose	+ ve
L-arabinose	+ ve

### Production procedure and chemical characterization of EPSS10

*Streptomyces* sp. MAE was grown in a medium designed to enhance the synthesis of EPS. To accomplish partial purification and fractionation, the EPS was redissolved in deionized water and dialyzed with deionized water for 72 h. To precipitate the dialyzed solution, four liters of cold ethanol were utilized. The isolated EPS, referred to as EPSS10, is characterized by a grayish-white color, an amorphous structure, and a lack of odor.

**Fig. 3:**
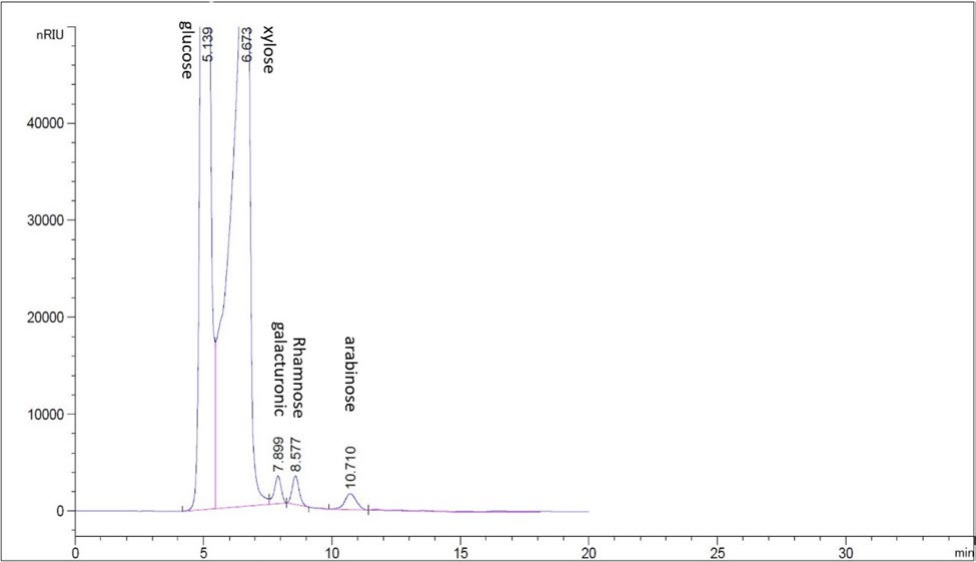
HPLC of EPSS10

The purified polysaccharide underwent DEAE-cellulose column chromatography and was eluted using deionized water, followed by 0.1 M NaCl, 0.3 M NaCl, and 0.5 M NaCl. The carbohydrate concentrations in each 15 ml aliquot of eluate were assessed. Further elution was only conducted if the final sample from the preceding elution contained no carbohydrates. EPSS10 contained uronic acid (19.55%). Therefore, the fraction had sulfate (25.03%). While N-acetyl glucose amine (10.63%). Molar ratio was determined using HPLC, showing that the EPSS10 fraction contained monosaccharide composition. Glucose: Rhamnose: Galacturonic acid: Xylose: Arabinose with a molar ratio of 2.0:1.0:1.0:0.5:0.5. this means the fraction was acidic heteropolysaccharides (Fig. 3).

**Fig. 4:**
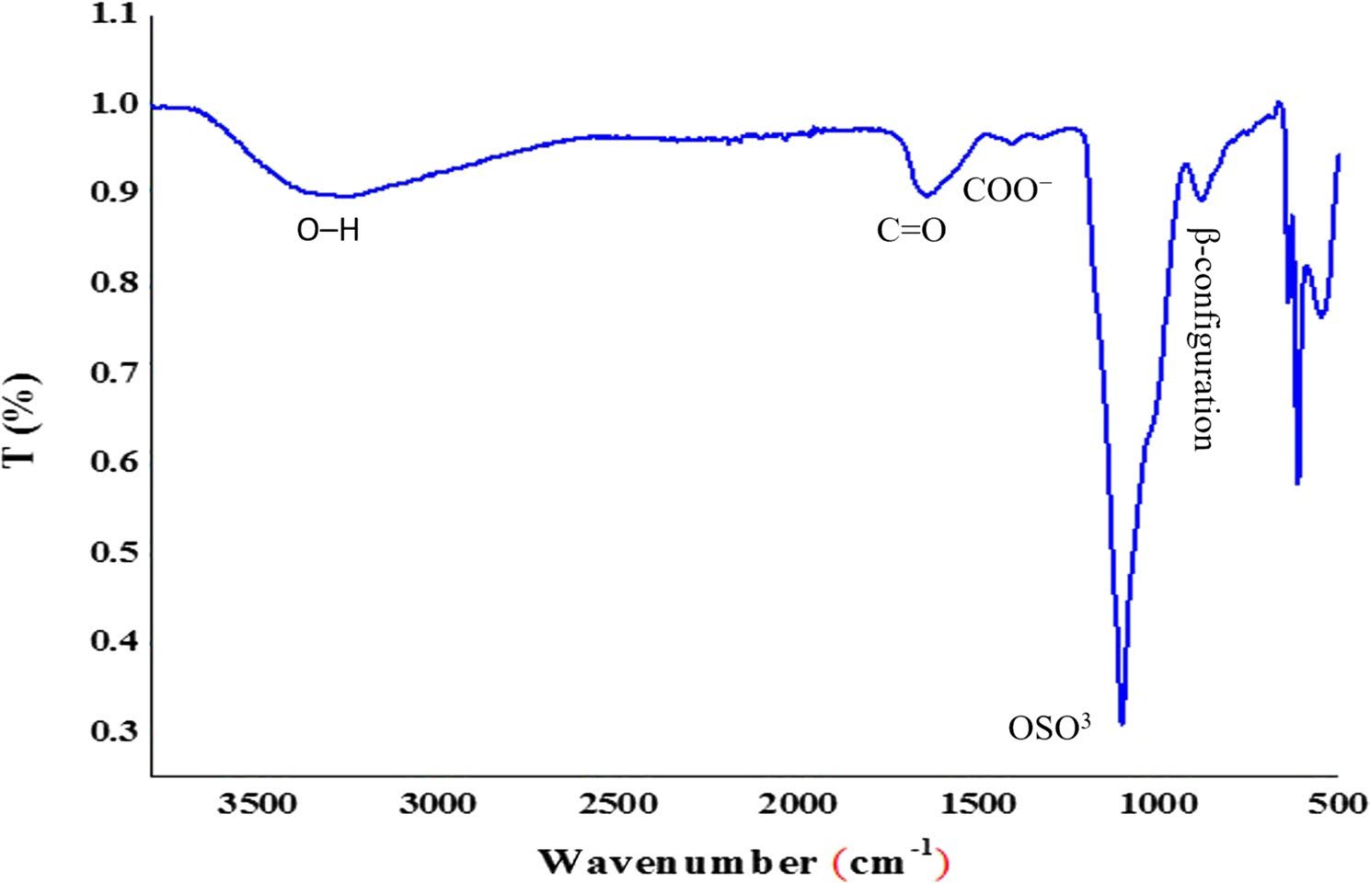
FTIR spectrum and functional groups of EPSS10

The functional groups of EPSS10 were characterized through FTIR spectroscopy, as illustrated in Fig. 4. A broad peak observed at approximately 3280.21 cm^−1^ corresponds to the OH stretching vibration of the polysaccharide [44], which falls within the range of 3600–3200 cm^−1^. The peaks observed at 1642.58 cm^−1^ are attributed to a C = O stretching vibration of the N-acetyl group or the protonated carboxylic acid in EPSS10, confirming the presence of uronic acid. Additionally, the absorptions near 1101.29 cm^−1^ are indicative of sulfate group, while the peak at 877.93 cm^−1^ suggests the presence of β-pyranose [45]. Consequently, the analysis conducted through infrared spectrometry indicated a strong likelihood that the EPSS10 was classified as a β-type heteropolysaccharide [46].

### In-vivo toxicity evaluation of EPSS10

**Fig. 5:**
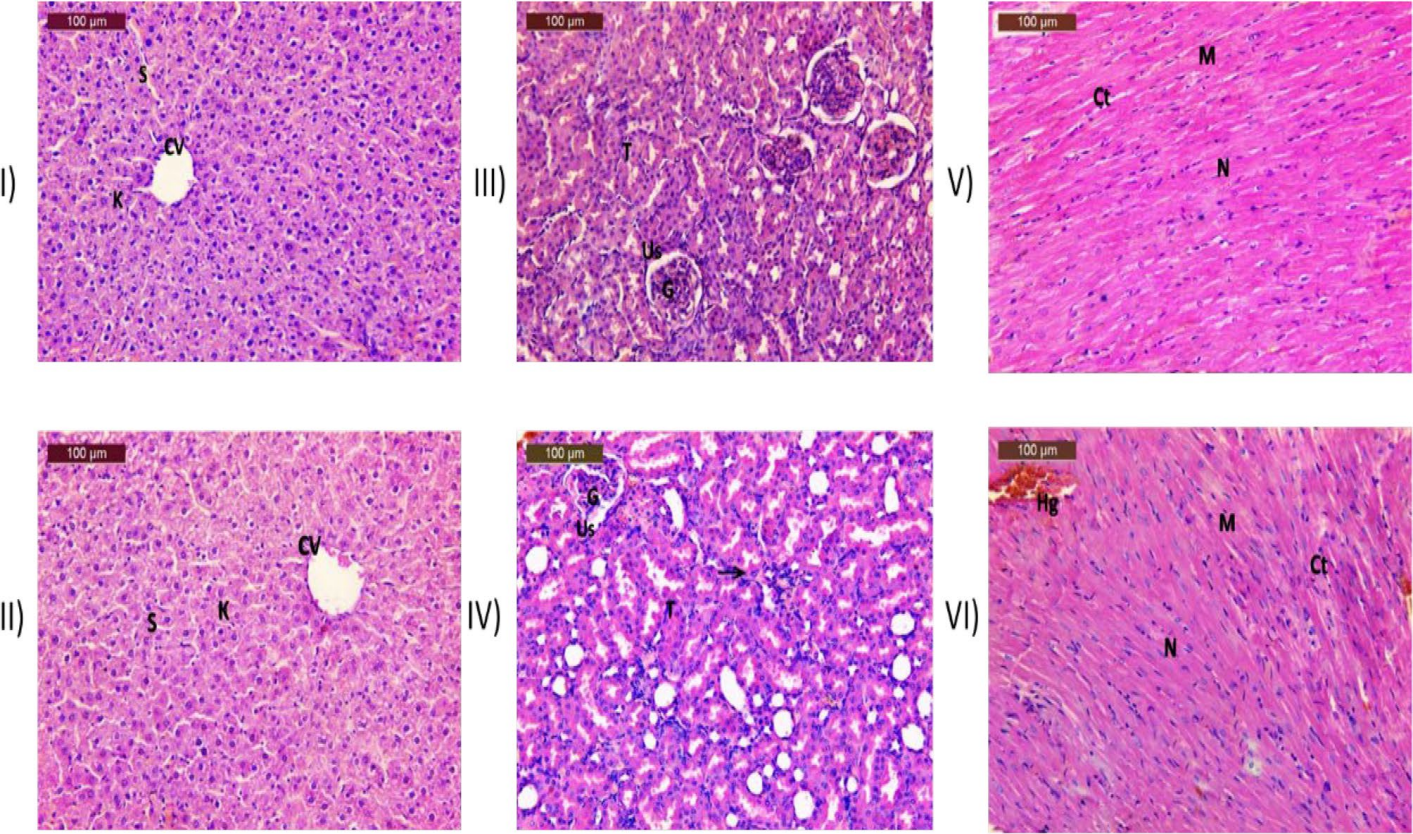
Histopathological toxicity study effect of EPSS10 on some rats' organs. The photomicrograph of group (**I**) depicts the liver from the control, group (**II**) showed liver treated with EPS showing normal reappearance of most hepatocyte, normal of central vein (Cv) and separated by sinusoids (s) with very few Kupfer cells (K). Group (**III**) demonstrates a control kidney section. group (**IV**) showed the kidneys from the EPS-treated reveal a largely normal restoration of the glomerulus (G), urinary space (Us), and renal tubules (T), although a small number of inflammatory cells are present (indicated by an arrow). group (**V**) showed heart section control. group (**VI**) showed heart section treated with EPSS10, the histological structure of the cardiac myocytes (M) and nuclei (N) appeared nearly normal, exhibiting only mild interstitial hemorrhage (Hg). Most myofibers were intact (Ct)

The evaluation of in vivo safety would involve the examination of blood elements and biochemical parameters, as presented in Table 2. Venous blood was obtained from the rats using heart punctures at the conclusion of the experiment. The reports showed non-significant results between all groups treated with EPSS10 and the control groups. The histopathological findings are presented in Fig. 5.The photomicrograph depicts the liver from the control group (I), highlighting normal hepatocytes (H) radiating from the central vein (CV) and separated by sinusoids (s). For the liver group (II) treated with EPS showing normal reappearance of most hepatocyte, normal of central vein (Cv) had very few Kupffer cells (K). Group (III) demonstrates a control kidney section characterized by a normal histological structure, featuring a typical renal corpuscle that includes the glomerular tuft (G) and is encircled by the urinary space (Us). The renal tubules are intact and exhibit a vascular nucleus (T). In contrast, the kidneys from the EPS-treated group (IV) reveal a largely normal restoration of the glomerulus (G), urinary space (Us), and renal tubules (T), although a small number of inflammatory cells are present (indicated by an arrow). Heart section control group (V) exhibited a typical histological structure of cardiac myocytes (M), with the majority arranged longitudinally and featuring centrally positioned, rounded vesicular nuclei (N). A delicate layer of connective tissue (CT) was found interspersed among the cardiac myocytes. In the group (VI) treated with EPSS10, the histological structure of the cardiac myocytes (M) and nuclei (N) appeared nearly normal, exhibiting only mild interstitial hemorrhage (Hg). Most myofibers were intact (Ct).

**Table 2:** Toxicity effect of EPSS10 on biological functions of rat models

		Before treatment	After treatment
Complete Blood Count	RBCs(×10^6^/µL)	5.39 ± 0.17	5.90 ± 0.59
	Hb (g/dL)	12.12 ± 0.27	12.98 ± 0.16
	Hct (%)	32.89 ± 0.89	35.90 ± 1.45
	MCV (fL)	66.34 ± 0.88	64.90 ± 2.52
	MCH (pg)	19.09 ± 0.28	20.71 ± 0.55
	MCHC (g/dL)	32.97 ± 1.90	34.86 ± 0.72
	Plt(×10^3^/µL)	673.8 ± 12.70	653.98 ± 52.07
	WBCs (×10^3^/µL)	5.4 ± 1.42	5.73 ± 1.64
Kidney Function	Creatinine (mg/dL)	0.6 ± 0.04	0.7 ± 0.06
	Urea (mg/dL)	39.03 ± 2.24	42.75 ± 2.11
Liver Function	ALT (IU/L)	59.32 ± 2.34	56.04 ± 4.19
	AST (IU/L)	39.53 ± 3.54	41.72 ± 2.97
	ALP (IU/L)	42.38 ± 3.18	49.87 ± 5.48
	Alb (g/dL)	3.72 ± 0.21	3.87 ± 0.28
	Total protein (g/dL)	5.53 ± 0.19	6.11 ± 0.19

All data are expressed as mean ± SD. T-test is used for data analysis (*n* = 3), to compare the means of all tested parameters before and after treatment. No Statistical significance was determined where *P value* > 0.05

### Antimicrobial activity of EPSS10

EPSS10 has significant antibacterial effects against Gram-positive (+) *S. aureus* NRRLB-767 and *B. subtilis* ATCC-6633 with 75.81 ± 0.37% and 79.02 ± 1.50%, respectively. Therefore, it has antibacterial activity against the Gram-negative bacteria *E. coli* ATCC-25,922 and *P. aeruginosa* ATCC-10,145 with 76.22 ± 0.87% and 74.99 ± 1.09%, respectively. It has no antifungal effect against *C. albicans* ATCC-10,231 and *A. niger* NRRLA-326, as shown in Table 3.

**Table 3:** Antimicrobial activity of EPSS10

	Antimicrobial activity (%)					
Compounds	Gram positive		Gram negative		Yeast	Fungi
	*S. aureus* NRRLB-767	*B. subtilis* ATCC 6633	*E. coli* ATCC 25,922	*P. aeruginosa* ATCC 10,145	*C. albicans* ATCC 10,231	*A. niger* NRRLA-326
EPSS10 (500 µg/ml)	75.81 ± 0.37	79.02 ± 1.50	76.22 ± 0.87	74.99 ± 1.09	- ve	- ve
Ciprofloxacin (20 µg/ml)	96.90 ± 0.86	91.63 ± 0.55	98.40 ± 0.22	98.87 ± 0.14	ND	ND
Nystatin (20 µg/ml)	ND	ND	ND	ND	97.27 ± 0.34	98.27 ± 0.11

### Antioxidant activity of EPSS10

**Fig. 6:**
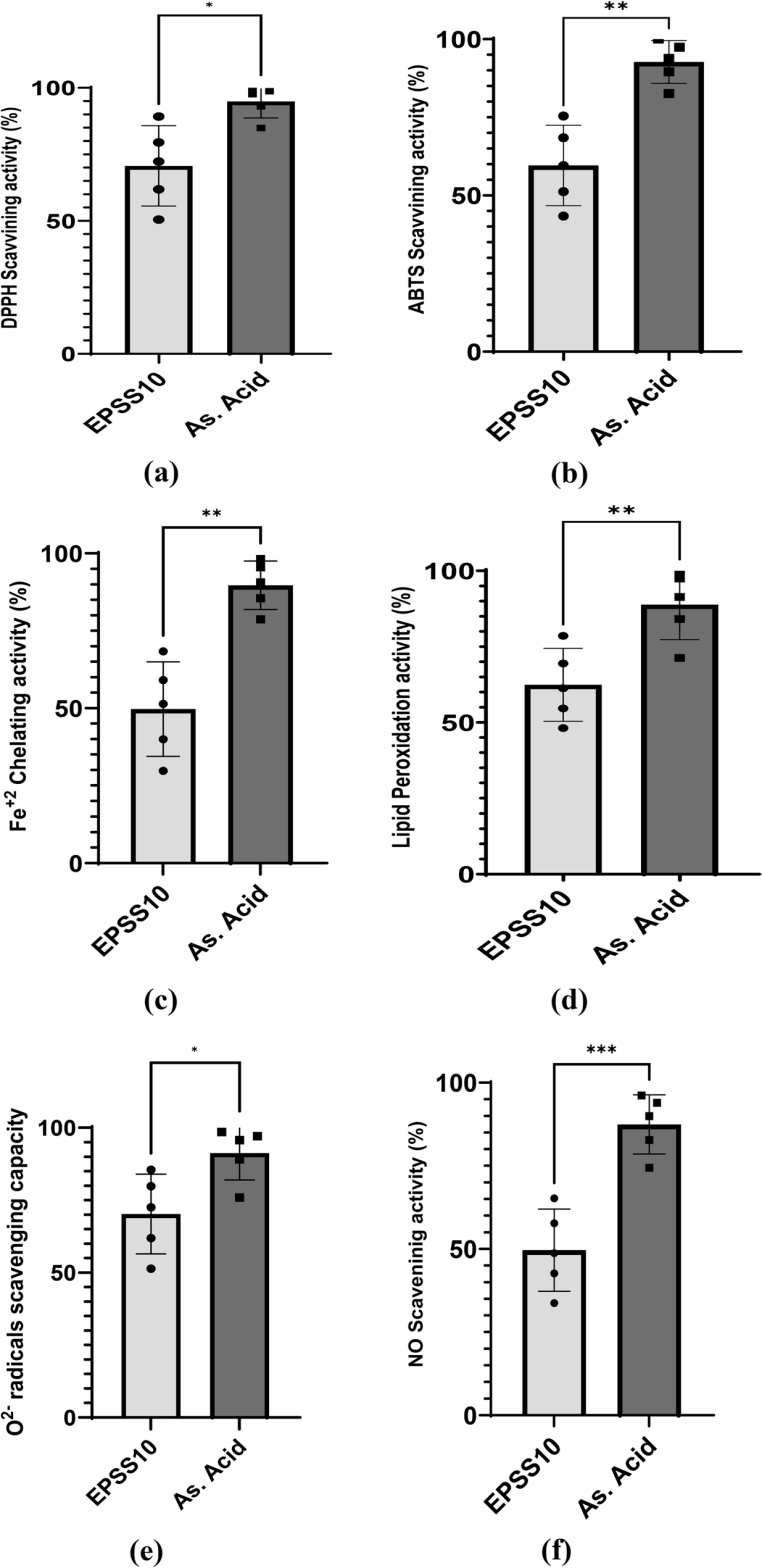
Antioxidant activity of EPSSS10 at different concentrations (100 - 500 μg/mL). Ascorbic acid (As. Acid) used as reference standard. All Data are presented as mean ± SEM. Welch's t test was used for data analysis (n = 3). Statistical significance is represented as follows: * p < 0.05, ** p < 0.001, ** p < 0.0001. **a** DPPH scavenging activity Inhibition percentage (%) **b** ABTS scavenging activity Inhibition percentage (%) **c** Fe+2 ion chelation ability Inhibition percentage (%) **d** Lipid peroxidation Inhibition percentage (%) **e** O2- radicals scavenging capacity Inhibition percentage (%) **f** NO scavenging capacity Inhibition percentage (%)

The quantitative assessment of DPPH free radical scavenging activity was conducted at various concentrations of EPSS10, specifically at 100, 200, 300, 400, and 500 µg/ml. The maximum antioxidant activity of EPSS10 was 89.21 ± 0.3% at 500 µg/ml (Fig. 6a). The capacity of EPSS10 to scavenge ABTS radicals at various concentrations is illustrated in Fig. 6b. At the lowest concentration of 100 µg/ml, EPSS10 exhibited a scavenging activity of 43.36 ± 1.28%, which progressively increased to 75.4 ± 0.5% as the concentration rose to 500 µg/ml. To assess the chelating efficiency of EPSS10, the chelation of ferrous ions (Fe+) among transition metals was analyzed by forming complexes with ferrozine, and the results are illustrated in Fig. 6c. EPSS10 exhibited a chelating percentage of 29.74 ± 0.4% at the minimum concentration, which increased to 68.32 ± 1.0% at the maximum concentration of 500 µg/mL. Consequently, the efficacy of EPSS10 in mitigating lipid peroxidation was evaluated via the thiocyanate method.

EPSS10 prevented the peroxidation of linoleic acid in a concentration-dependent manner, exhibiting the least inhibitory effect at lower concentrations (48.09 ± 0.5%) seen at the minimum concentration (100 µg/ml) as depicted in Figure (6d). The highest percentage (78.57 ± 1.2%) was obtained at the maximal concentration (500 µg/ml). Figures (6e) showed the percentage of superoxide anion (SOR) production that was suppressed by EPSS10 at varying concentrations. EPSS10 exhibited SOR scavenging percentages ranging from 51.4 ± 1.0% at 100 µg/ml to 85.5 ± 0.940% at 500 µg/ml. Also, the NO radical scavenging capacity of EPSS10 was assessed by an SNP-generating NO system. Data presented in Figure (6f); EPSS10 exhibited a notable reduction in nitrite released in the SNP test medium, indicating moderate nitric oxide scavenging activity. The NO scavenging activity of EPSS10 increased dramatically from 33.8 ± 1.0% at 100 µg/ml to 65.2 ± 0.6% at the maximum dosage of 500 µg/ml.

### Anti-inflammatory activity of EPSS10

**Fig. 7:**
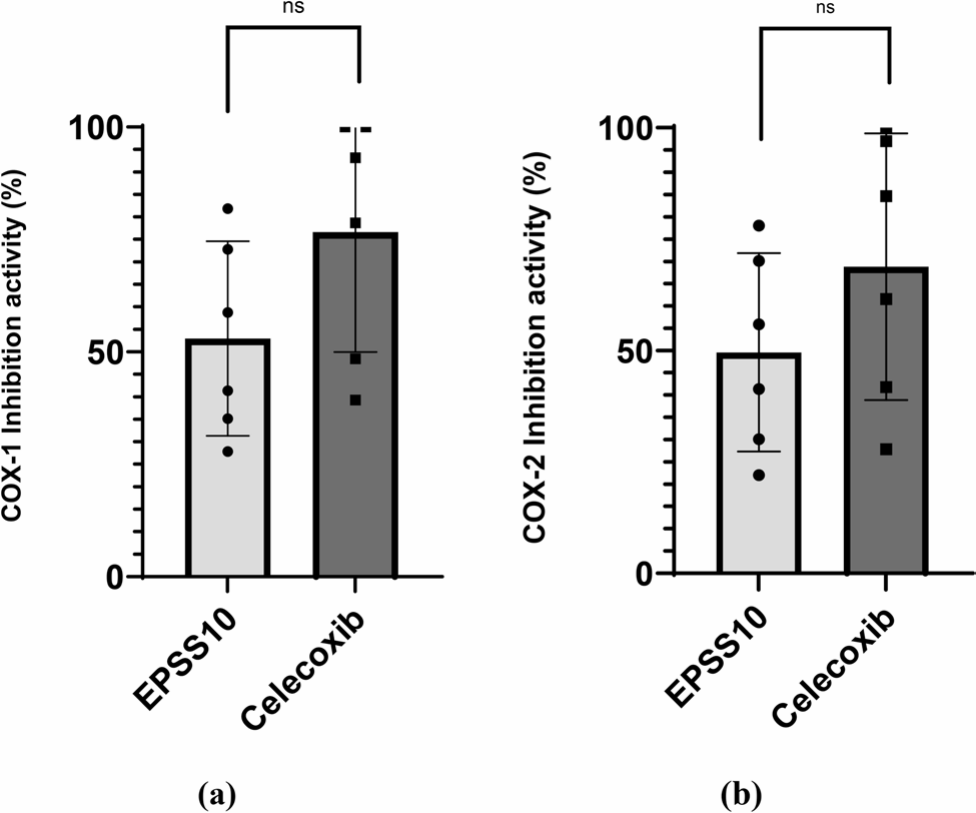
COX-1 and COX-2 inhibition by EPSS10 and standard drug (Celecoxib). All Data are presented as mean ± SEM. Welch's t test was used for data analysis (n = 3). Statistical significance is represented as follows: ns: non-significant (p >0.05). **a **COX-1 inhibition activity Percentage (%) **b **COX-2 inhibition activity Percentage (%)

The anti-inflammatory activity of EPSS10 was assessed through various methods, such as COX-1 inhibitory, and showed that the inhibition percentage was 81.9 ± 0.5% and IC_50_ was 22.80 µg/ml, while the control sample (Celecoxib) gave IC_50_ was 8.71 µg/ml. so, the COX-2 inhibitory provided 78.0 ± 1.2% and IC_50_ was 27.57 µg/ml, while the control (Celecoxib) gave IC_50_ was 12.1 µg/ml (Fig. 7a, b).

### Antitumor activity against different cell lines

**Fig. 8:**
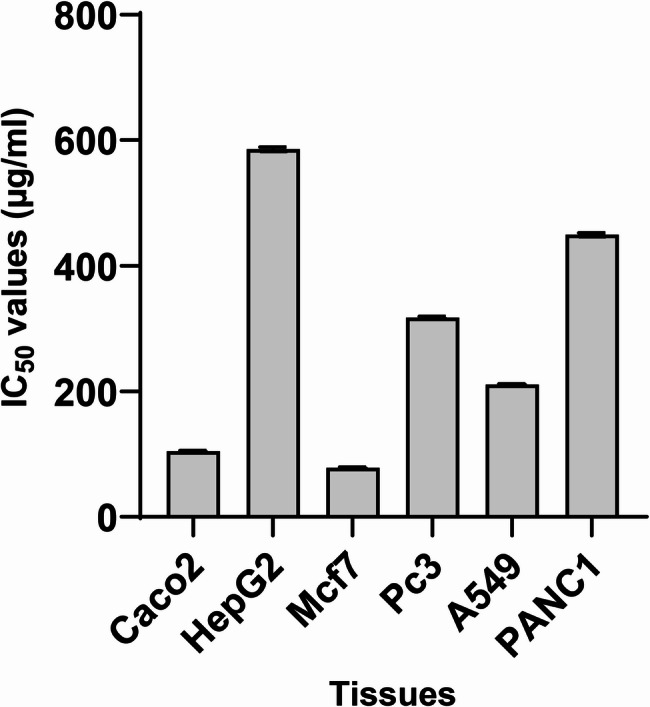
Anti-cancer activity of EPSS10 against several cell lines, (HepG2) liver cancer, (CaCo2) colon cancer, (MCF7) breast cancer, (A549) lung cancer, (PC3) prostate cancer, and (PANC1) pancreatic cancer

Antitumor activity was evaluated by the MTT viability/cytotoxicity method against HepG2, CaCo2, MCF7, PC3, A549, and PANC1 cell lines. The fraction EPSS10 had a cytotoxic effect on different cell lines; by increasing the concentrations, the viability gradually decreased. IC_50_ values were calculated to be 585.51 ± 3.33, 104.39 ± 1.24, 78.12 ± 0.86, 317.33 ± 1.92, 210.83 ± 0.72 and 449.31 ± 2.91 µg/ml for cell lines HepG2, CaCo2, MCF7, PC3, A549 and PANC1, respectively, as shown in Figure (8) & Supplementary Figures (1–6).

### Real-time PCR

**Fig. 9:**
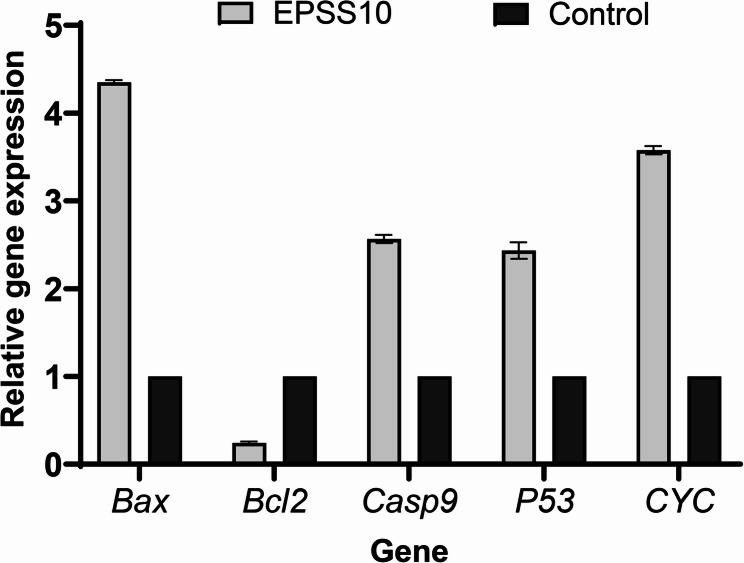
Gene expression profile for target genes over a 24-h treatment period was determined by real-time PCR. The fold change in expression was calculated relative to control values (without EPSS10) with GAPDH as the internal control using the 2^(-ΔΔCT) method. Control values were calculated using ∆Ct values form control cells and tested cells by using target gene expression to GAPDH gene. Results are meaning three experiments

According to Fig. 9, the levels of *Bax*,* Bcl2*,* p53*,* Cytochrome c* (*CYC*), and *Caspase 9* (*Casp 9*) in cells treated with EPSS10 were assessed to investigate the potential mechanism of EPSS10 as an anti-cancer effect on MCF7 cells. The expression level of the *Bax* gene increased by 4.3-fold compared to the control after treatment with EPSS10, while Bcl2 was decreased to 0.3-fold. In contrast, the expression of the Casp *9* gene was found to be elevated by 2.5-fold in comparison to the control group. Additionally, treatment with EPSS10 resulted in a 1.6-fold increase in the expression level of the *p53* gene. Furthermore, the expression of *CYC* rose significantly after EPSS10 treatment, reaching 3.5-fold the control level 24 h post-treatment.

### Antiviral activity against the hepatitis A virus (HAV)

**Fig. 10:**
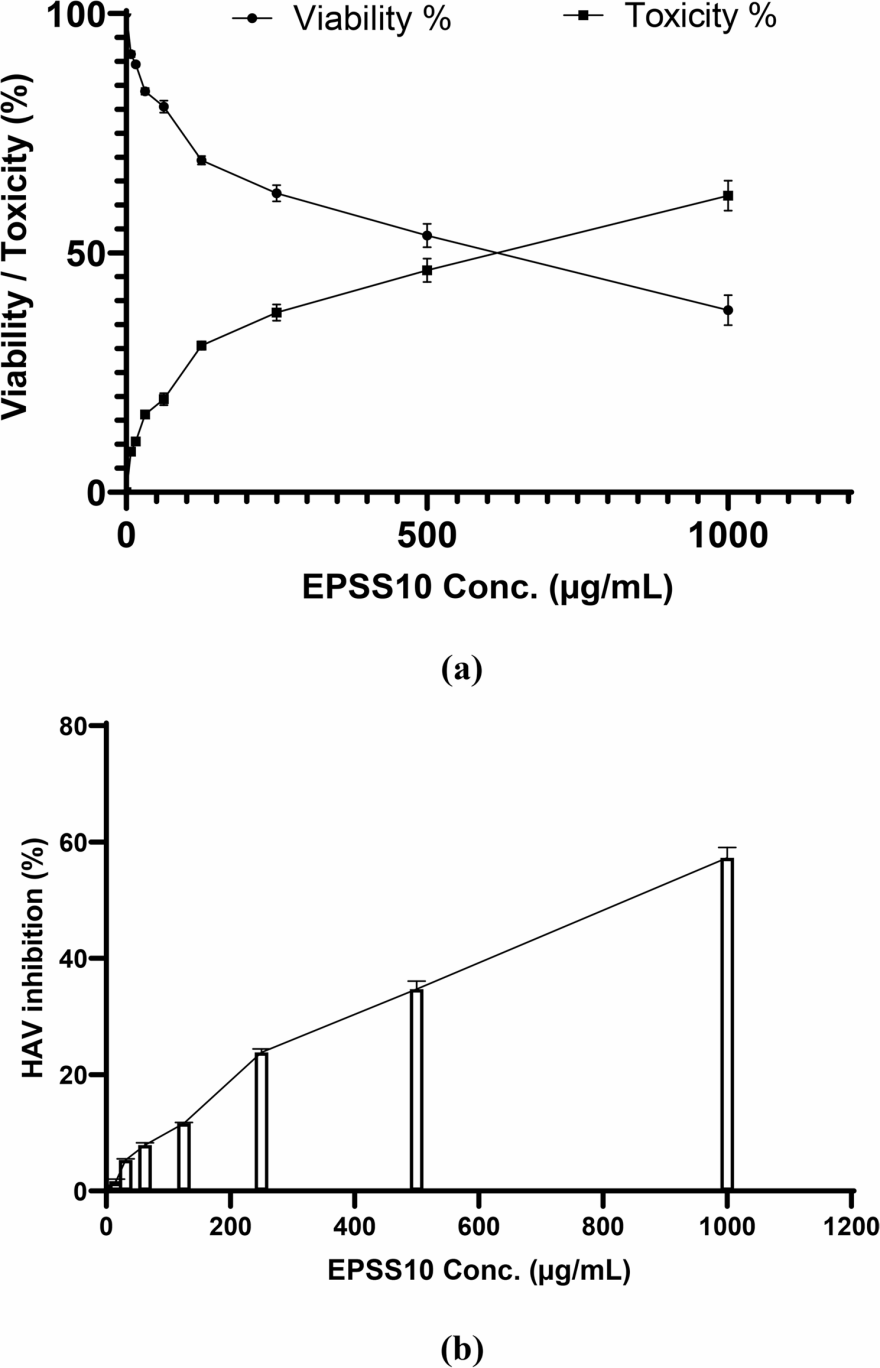
**a** Effect of EPSS10 on Vero cells at different concentration & (**b**) antiviral activity of EPSS10 against HAV

Using the MTT viability/cytotoxicity assay, the maximum nontoxic concentration (MNTC) was determined to be the highest dose used in antiviral activity determination, whereas the MNTC of the EPSS10 was 1000 µg/mL. Therefore, different concentrations (1000, 500, 250, 125, 62.5, 31.25, 15.6, and 7.8 µg/mL) from EPSS10 were used for evaluation of antiviral activity against HAV. Half maximal cytotoxic concentration (CC_50_) was 616.40 ± 27.91 µg/mL as shown in Fig. 10a. Results illustrated that the antiviral activity of EPSS10 was 57.29 ± 1.78 at MNTC and the half maximal viral cytopathogenicity inhibitory concentration (EC_50_) of EPSS10 was 838.50 ± 34.62 µg/mL against HAV as shown in Figure (10b).

## Discussion

EPSs derived from marine microorganisms are pertinent to developing new pharmaceutical compounds; however, there are limited reports concerning EPSs exhibiting antioxidant, anticancer, and antiviral activities isolated from marine microorganisms [10]. EPSs obtained from diverse sources and through various chemical processes exhibit a range of chemical compositions, molecular weights, and structural characteristics. EPS possess distinct physicochemical properties such as gelation, solubility, minimal osmotic effects, and surface characteristics, which are influenced by their composition and processing methods [47]. More research is also required to thoroughly understand the mechanisms of action and determine which EPSs are most promising for therapeutic uses. In our study, we isolated streptomycetes from marine sources from Hurghada. To achieve high EPS productivity, 12 streptomycetes isolates were randomly selected and evaluated for EPS production in a particular production medium. S10 isolate may provide the highest EPS productivity (7.6 g/L), while, according to Mohamed et al. [23] *Streptomyces rochie* produced 14.2 g/L of EPS. Then, S10 was identified biochemically, morphologically, and physiologically. Phylogenetic analysis was conducted using the sequence of the 16 S rRNA gene for strain S10 and identified as *Streptomyces* sp. MAE under accession number OR354383, as in a similar study by Mohamed et al. [23], who identified the promising *Streptomyces* isolate that produced EPS based on molecular identification.

The main fraction (EPSS10) contained 19.55% uronic acid. The proportion of sulfate was 25.03%. The N-acetyl glucose amine, however, was 10.63%. Molecular ratio was determined using HPLC showed that the EPSS10 fraction contained monosaccharide composition Glucose: Rhamnose: Galacturonic acid: Xylose: Arabinose with a molar ratio 2.0:1.0:1.0:0.5:0.5 respectively, this means the fraction was acidic heteropolysaccharides. However, EPS was found to be made up of mannuronic acid, glucuronic acid, xylose, and fructose at a molar ratio of 1.0: 0.5: 1.0: 2.0, respectively, according to the mono sugars in another study by Mohamed et al., [23]. Moreover, EPS with uronic and sulfate groups enhanced its antioxidant and anticancer properties [6].

The structural characteristics of polysaccharides, such as their chemical composition, molecular weight, types of glycosidic bonds, and conformation, significantly affect their bioactivity. Divergences in source materials, extraction methods, and drying techniques that affect physiochemical properties will result in variations in antioxidant activity. A well-documented relationship exists between molecular weight and radical scavenging activity [48]. Furthermore, it is proposed that the quantity of hydroxyl or ionic groups in polysaccharides contributes to their overall radical scavenging capacity [49]. Compounds that feature the functional groups -OH, -SH, -COOH, -PO_3_H_2_, -C = O, -NR_2_, -S-, and -O- show a strong capacity for chelation. Consequently, the presence of uronic acid and sulfate groups is crucial for exhibiting the metal-chelating properties of polysaccharides.

Antioxidant activity of EPSS10 was conducted using several methods (DPPH, ABTS, Fe^2+^ ion chelation ability, Lipid peroxidation Inhibition capacity, O^2−^ radicals scavenging capacity, and NO scavenging capacity). Radical scavenging mechanisms by antioxidants can be categorized into two primary processes: hydrogen atom abstraction and radical adduct formation (RAF). Hydrogen atom abstraction can occur via three common mechanisms: hydrogen atom transfer (HAT), electron transfer followed by proton transfer (ETPT), and sequential proton loss electron transfer (SPLET) [50]. According to our results, EPSS10 had a high DPPH scavenging potential that increased with EPSS10 concentration. Evidently, 500 µg/ml of EPSS10 had the highest antioxidant activity (89.21 ± 0.3%). The results of antioxidant activities of EPSS10 obtained in this work were consistent with Khalil et al., [51], who found that the radical scavenging capacity of Exopolysaccharides (EPS1 and EPS5) was 77% and 75.2%, respectively, and considerably contributing to antioxidant activity. Also, These outcomes are consistent with those of Ghanaim et al. [52]., who found that EPS derived from *Pseudomonas aeruginosa* AG01 had antioxidant activity. The IC_50_ values for DPPH radical and ABTS scavenging with partially purified EPS were found to be 218.30 µg/ml and 293.77 µg/ml, respectively. The study of Hazra et al. [53] furnishes evidence that the EPS demonstrated significant antioxidant and free radical scavenging capabilities, as well as commendable reducing power, in vitro, relative to the conventional antioxidant, vitamin C. The EPS demonstrated significant antioxidant and free radical scavenging capabilities, as well as hydrogen peroxide scavenging and reducing capacity.

Inflammation, characterized by erythema, edema, dolor, and color, is an essential defense mechanism for the host against pathogens that invade. Pro-inflammatory stimuli, such as TNF-, initiate cellular reactions that enhance the synthesis of various cytokines, including prostaglandins (PGs) and nitric oxide (NO), as a response to inflammation [54]. Inflammatory diseases are caused by the overabundance of mediators involved in the arachidonic acid (AA) cascade, particularly the lipoxygenase (LOX) and cyclooxygenase (COX) pathways. The pro-inflammatory enzymes, COX-1 and COX-2, are the two isoforms of COX, and they are identical in structure and catalytic activity. EPSS10 revealed significant anti-inflammatory activity. The EPSS10 exhibited inhibition of COX-1 (22.80 µg/mL) and COX-2 (27.57 µg/mL), compared to the standard drug celecoxib (8.71 and 12.1 µg/mL, respectively). According to these results, EPSS10 may be used to treat inflammatory diseases. Presently, inflammation is often treated with non-steroidal anti-inflammatory drugs (NSAIDs). However, prolonged NSAID use can have negative consequences on kidney function, cardiovascular health, and the gastrointestinal tract. This finding offers NSAID substitutes that may have fewer adverse effects.

Microbial polysaccharides offer numerous advantages compared to plant- or animal-derived polysaccharides, including precise and consistent production parameters, as well as superior yield and quality of the final product [55, 56]. Numerous studies have indicated that EPSs can impede cancer proliferation through the following mechanisms: (i) prevention of tumorigenesis through oral intake of active formulations; (ii) direct anti-cancer effects, including the induction of apoptosis in cancer cells; (iii) immunopotentiation in conjunction with chemotherapy; and (iv) inhibition of tumor metastasis [57–59]. This study looks into the anticancer qualities of EPSS10 as a possible cancer treatment. The cytotoxicity of EPSS10 was evaluated using the MTT viability/cytotoxicity method against HepG2, CaCo2, MCF7, PC3, A549, and PANC1 cell lines. Treatment with EPSS10 produced different cellular responses, according to the results of the cytotoxicity tests conducted in this work. EPSS10 exhibited the lowest IC_50_ values on CaCo2, MCF7, and A549 (104.39 ± 1.24, 78.12 ± 0.86, and 210.83 ± 0.72 µg/ml), respectively. However, EPS5 derived from lactic acid bacteria (LAB) had a cytotoxic effect on MCF7, CaCo2, and HepG2 with IC_50_ values of 7.91, 10.69, and 9.12 mg/ml, respectively [51]. Furthermore, EPS produced from *Bacillus* sp. has a strong potential for cytotoxicity against MCF7 cells at low concentrations [60]. Selim et al. [61] reported the production of an EPS from *Streptomyces carpaticus*, which exhibited significant cytotoxic effects, achieving 51.7% and 59.1% inhibition against breast and colon tumor cell lines, respectively. A chemical investigation of this EPS revealed its monosaccharide composition and molecular weight (Mw) to be 1.180 × 10^5 g/mol. Mohamed et al. [62] isolated an EPS obtained from *Bacillus altitudinis* containing mannuronic acid and glucose with a molecular ratio of 1:2.2 with Mw 4.23 × 10^5^ Da. The EPS demonstrated notable antitumor effects against Ehrlich Ascites carcinoma cells and the A-549 lung cancer cell line, exhibiting an IC_50_ value of 51.94 µg/ml.

In the same context, Ghanaim et al. [52] isolated EPS from *Pseudomonas aeruginosa* AG01 that had anticancer activity against prostate cancer (PC3) and breast cancer (MCF7) cells with IC_50_ values of 156.41 and 156.41 µg/ml, respectively. EPS resulted in apoptosis induction in MCF7 cells.

Because of the anticancer evidence, we looked into how EPSS10, which is produced from *Streptomyces* sp. MAE, affected the expression of apoptotic marker genes in MCF7 cells. In this investigation, we measured the relative expression of the apoptotic genes *Bax*,* Bcl2*,* P53*,* Cytochrome c (CYC)*, and *Caspase-9*.

Researchers examined the mechanism by which EPSS10 causes apoptosis in cancerous cells using *Bcl2* family proteins, including *Bcl2*, which prevents apoptosis, and *Bax*, which causes apoptosis. According to the gene expression analysis's findings, EPSS10 raised the expression of the *Bax* gene in the cell lines under investigation. However, *Bcl2* gene expression was significantly reduced in cells treated with EPSS10. This finding agrees with the study of Khalil et al. [51] who showed that EPS5 obtained from *Lactobacillus delbrueckii* ssp. Bulgaricus DSM 20081 T was found to enhance the expression of the genes *Bax*, *Caspase 8*,* Caspase 3*, and *p53*. Conversely, there was a decrease in the expression of the *Bcl2*, MCL1, and *Vimentin* genes. *Caspase*−9 is an initiating *caspase* that is essential for mitochondria-mediated apoptosis. Our findings showed a substantial overexpression of *Caspase* 9 in treated MCF7 cells relative to controls. According to Tukenmez et al. [63], *Lactobacillus delbrueckii* ssp. bulgaricus B3-EPS increased the expression of *Bax*, *Caspase* 3, and *Caspase* 9 while lowering that of *Bcl2*, which hindered time-dependent proliferation and caused apoptosis.

Additionally, pro-apoptotic genes associated with both intrinsic and extrinsic pathways can be regulated by the *p53* tumor suppressor [64]. In the MCF7 cell line under test, EPSS10 raised the amount of *p53* mRNA, suggesting that a *p53*-dependent pathway is involved in the MCF7 cells' apoptotic process. El-Deeb et al. [65] found that *p53* gene expressions rose following treatment of CaCo2 cells with *L. acidophilus* LA-EPS-20,079 EPS protein called *cytochrome c* is involved in both programmed cell death and cellular respiration. On the other hand, *cytochrome c* is released from the mitochondria during apoptosis, which sets off a series of events that ultimately result in cell death. In our study, *cytochrome c* (*CYC*) in MCF7 cells was up-regulated.

Board proteins, including *Bcl2*, control cell division and damage. Because they preserve the integrity of the mitochondrial membrane, *Bcl2* family proteins are necessary for mitochondria-mediated apoptosis. Flow cytometry analysis revealed that SPS, a new polysaccharide extracted from *Sargassum integerrimum*, can trigger cellular apoptosis, reduce mitochondrial membrane potential (MMP), produce reactive oxygen species (ROS), and induce G2/M phase arrest in the cell cycle of A549 cells. Alongside the increased expression of *p53* and *Bax*, SPS treatment also results in decreased *Bcl2* expression and the activation of cleaved *caspase* 3, *caspase* 9, and PARP. SPS suppresses the growth, movement, and cord formation of umbilical vein endothelial cells (HUVECs) in vitro, and it also hinders angiogenesis in zebrafish embryos in vivo [66].

Research on the antiviral properties of polysaccharides and their oligosaccharide derivatives is garnering heightened interest, with polysaccharides leading a novel trend in antiviral pharmaceuticals. Polysaccharides have the potential to impede viral replication by interfering with the viral life cycle or to enhance the host's antiviral immune responses. The life cycle of viruses differs considerably across various species; however, it consists of six essential stages: viral adsorption, entry, capsid uncoating, biosynthesis, viral assembly, and release.

The antiviral properties of polysaccharides encompass several mechanisms: (a) direct destruction of viruses, (b) prevention of viral attachment, (c) blockage of virus entry and uncoating, (d) suppression of viral transcription and replication, and (e) stimulation of the host's antiviral immune responses [48]. In this study, the EPSS10 fraction antiviral activity had been evaluated. Using the MTT viability/cytotoxicity assay, the maximum nontoxic concentration (MNTC) was determined to be the highest dose used in antiviral activity determination. The half maximum cytotoxic concentration (CC_50_) was 616.40 ± 27.91 µg/ml. At 1000 µg/ml, EPSS10 displayed a HAV inhibition percentage of 57.29 ± 1.78. EPSS10's half maximum viral cytopathogenicity inhibitory concentration (EC_50_) was 838.50 ± 34.62 µg/ml. By preventing viral adsorption, sulfated polysaccharides (ASWPH) isolated from *Aphanothece sacrum* show antiviral efficacy against Herpes Simplex virus type-2 (HSV-2) and Influenza A (H1N1), with an IC_50_ of 0.32–1.2 µg/mL [67]. EPS isolated from *Pseudomonas aeruginosa* AG01 had significant antiviral activity against both the Herpes Simplex virus type-1 (HSV-1) and the HAV [52].

EPS extracted from a newly isolated halotolerant cyanobacterium, *Acaryochloris* Al-Azhar MNE ON864448.1, demonstrated strong antiviral properties at various phases of HSV-1 and HSV-2, adenovirus (ADV), and coxsackievirus (A16) infections [68]. Cimino et al. [69] indicated that rosacelose, a novel anti-HIV polysaccharide made up of glucose and fucose sulfate, was extracted from the marine sponge *Mixylla rosacea*. The sulfuric acid group's presence is essential to polysaccharide virucidal action. This group interacts with viral proteins to improve a polysaccharide's virucidal qualities. The attraction between the negatively charged sulfate group and the positively charged amino groups present on the surface of viral proteins may be the cause of this interaction [70].

## Conclusion

The present study reports the production of the EPSS10 from *Streptomyces* sp. MAE, isolated from a marine sample collected in the Red Sea, Hurghada, Egypt. The in vivo toxicity assessment of EPSS10 in rats revealed that biological parameter analysis showed no significant differences between the EPSS10-treated groups and the control groups. EPSS10 demonstrated significant antibacterial activity. It exhibited antioxidant properties (DPPH, ABTS, ferrous reducing, nitric oxide scavenging, radical scavenging capability, and inhibition of lipid peroxide production) and selective inhibitory effects on COX-1 and COX-2, suggesting its potential as a natural raw material for inflammation treatment. Additionally, it showed the ability to fight cancer in several types of cells, including liver cancer (HepG2), colon cancer (CaCo2), breast cancer (MCF7), lung cancer (A549), prostate cancer (PC3), and pancreatic cancer (PANC1) by increasing the levels of *Bax*, *p53*, *Cytochrome* c, and *Caspase 9* to induce apoptosis. Moreover, it exhibited antiviral activity against the HAV. The structure and characteristics of EPSS10 may be responsible for its therapeutic properties.

## Supplementary Information

Supplementary Material 1.

## Data Availability

The data that support the findings of this study are available from the corresponding author upon reasonable request.

## Abbreviations

A549: Lung carcinoma
ABTS: 2,2'-azino-bis (3-ethylbenzothiazoline-6-sulfonic acid)
ALT: Alanine Transaminase
AST: Aspartate transferase
CaCo-2: Colon cancer cell line
COX: Cyclooxygenase
DPPH: 1,1-diphenyl-2-picrylhydrazyl
EPS: Exopolysaccharide
Hb: Hemoglobin
HepG2: Liver cancer cell line
HPLC: High-performance liquid chromatography
FT-IR: Fourier transform infrared spectroscopy
MCF7: Breast cancer cell line
NSAIDs: Non-steroidal anti-inflammatory medicines
PANC1: Pancreatic cancer cell line
PC3: Prostate carcinoma cells
RBCs: Red Blood Cells
ROS: Reactive oxygen species

## References

[1] Williams ST, Goodfellow M, Alderson G, Wellington EM, Sneath PH, Sackin MJ. Numerical classification of streptomyces and related genera. J Gen Microbiol. 1983;129:1743–813. 10.1099/00221287-129-6-1743. 6631406 10.1099/00221287-129-6-1743

[2] Abdalla AK, Ayyash MM, Olaimat AN, Osaili TM, Al-Nabulsi AA, Shah NP, et al. Exopolysaccharides as antimicrobial agents: mechanism and spectrum of activity. Front Microbiol. 2021;12:664395. 10.3389/fmicb.2021.664395. 34093478 10.3389/fmicb.2021.664395 PMC8170130

[3] El Awady M, Refat E, Nasr-Eldin M, Attia A. Effect of different parameters on the production of exopolysaccharides with antioxidant activity from marine streptomycetes. Benha J Appl Sci. 2023;8:23–30. 10.21608/bjas.2024.252692.1290.

[4] Salimi F, Farrokh P. Recent advances in the biological activities of microbial exopolysaccharides. World J Microbiol Biotechnol. 2023;39:213. 10.1007/s11274-023-03660-x. 37256348 10.1007/s11274-023-03660-x PMC10230149

[5] Kumari J, Kumawat R, Prasanna R, Jothieswari D, Debnath R, Ikbal AMA, et al. Microbial exopolysaccharides: classification, biosynthetic pathway, industrial extraction and commercial production to unveil its bioprospection: a comprehensive review. Int J Biol Macromol. 2025;297:139917. 10.1016/j.ijbiomac.2025.139917. 39824430 10.1016/j.ijbiomac.2025.139917

[6] Abdallah HMI, Abdel-Rahman RF, El Awdan SA, Allam RM, El-Mosallamy AEMK, Selim MS, et al. Protective effect of some natural products against chemotherapy-induced toxicity in rats. Heliyon. 2019;5:e01590. 10.1016/j.heliyon.2019.e01590. 31080906 10.1016/j.heliyon.2019.e01590 PMC6507045

[7] Abdelhamid SA, Mohamed SS, Selim MS. Medical application of exopolymers produced by marine bacteria. Bull Natl Res Cent. 2020;44:69. 10.1186/s42269-020-00323-x.

[8] Abdel-Monem MO, Hassan MG, El Awady ME, Moharam BA, Mohamed SS. Production and optimization of exopolysaccharides with antioxidant activity isolated from marine bacteria. Benha J Appl Sci. 2022;7:207–13. 10.21608/bjas.2022.257797.

[9] Abdelhamid SA, Mohamed SS, Abo Elsoud MM, Selim MS, Mounier MM, Eltaher A, et al. Characterization and modeling of marine Bacillus cereus strain MSS1 exopolysaccharide and its antagonistic effect on colon cancer. Probiotics Antimicro Prot. 2025. 10.1007/s12602-025-10539-w. 10.1007/s12602-025-10539-w PMC12999837 40320507

[10] Y RS AYK. A comprehensive review of anticancer, immunomodulatory and health beneficial effects of the lactic acid bacteria exopolysaccharides. Carbohydr Polym. 2019;217. 10.1016/j.carbpol.2019.04.025. 10.1016/j.carbpol.2019.04.025 31079688

[11] Lunar MM, Markočič P, Fujs Komloš K, Štamol T, Poljak M. Sporadic hepatitis A, virus PCR. False-positive results observed during reflex testing of serum samples previously tested for Anti-HAV antibodies and caused by contamination with HAV RNA present in the reagents of the commercial Anti-HAV immunoassay. Microbiol Spectr. 2023;11:e0012223. 10.1128/spectrum.00122-23. 37162362 10.1128/spectrum.00122-23 PMC10269866

[12] Bray F, Laversanne M, Weiderpass E, Soerjomataram I. The ever-increasing importance of cancer as a leading cause of premature death worldwide. Cancer. 2021;127:3029–30. 10.1002/cncr.33587. 34086348 10.1002/cncr.33587

[13] Jurášková D, Ribeiro SC, Silva CCG. Exopolysaccharides produced by lactic acid bacteria: from biosynthesis to health-promoting properties. Foods. 2022;11:156. 10.3390/foods11020156. 35053888 10.3390/foods11020156 PMC8774684

[14] Hou C, Yin M, Lan P, Wang H, Nie H, Ji X. Recent progress in the research of *Angelica sinensis* (Oliv.) Diels polysaccharides: extraction, purification, structure and bioactivities. Chem Biol Technol Agric. 2021;8:13. 10.1186/s40538-021-00214-x.

[15] Koller VJ, Marian B, Stidl R, Nersesyan A, Winter H, Simić T, et al. Impact of lactic acid bacteria on oxidative DNA damage in human derived colon cells. Food Chem Toxicol. 2008;46:1221–9. 10.1016/j.fct.2007.09.005. 17942208 10.1016/j.fct.2007.09.005

[16] Angelin J, Kavitha M. Exopolysaccharides from probiotic bacteria and their health potential. Int J Biol Macromol. 2020;162:853–65. 10.1016/j.ijbiomac.2020.06.190. 32585269 10.1016/j.ijbiomac.2020.06.190 PMC7308007

[17] Wu Z, Wang G, Pan D, Guo Y, Zeng X, Sun Y, et al. Inflammation-related pro-apoptotic activity of exopolysaccharides isolated from *Lactococcus lactis* subsp. *Lactis*. Beneficial Microbes. 2016;7:761–8. 10.3920/BM2015.0192. 27459336 10.3920/BM2015.0192

[18] Hayakawa M, Nonomura H. Humic acid-vitamin agar, a new medium for the selective isolation of soil actinomycetes. J Ferment Technol. 1987;65(5):501–9. 10.1016/0385-6380(87)90108-7.

[19] Waksman SA. The Actinomycetes. Vol. II. Classification, identification and descriptions of genera and species. The Actinomycetes. Vol II Classification, identification and descriptions of genera and species. 1961.

[20] Gardes M, Bruns TD. ITS primers with enhanced specificity for basidiomycetes - application to the identification of mycorrhizae and rusts. Mol Ecol. 1993;2:113–8. 10.1111/j.1365-294X.1993.tb00005.x. 8180733 10.1111/j.1365-294x.1993.tb00005.x

[21] Manivasagan P, Sivasankar P, Venkatesan J, Senthilkumar K, Sivakumar K, Kim S-K. Production and characterization of an extracellular polysaccharide from streptomyces Violaceus MM72. Int J Biol Macromol. 2013;59:29–38. 10.1016/j.ijbiomac.2013.04.012. 23597709 10.1016/j.ijbiomac.2013.04.012

[22] Liu C, Lu J, Lu L, Liu Y, Wang F, Xiao M. Isolation, structural characterization and immunological activity of an exopolysaccharide produced by *Bacillus licheniformis* 8-37-0-1. Bioresour Technol. 2010;101:5528–33. 10.1016/j.biortech.2010.01.151. 20199860 10.1016/j.biortech.2010.01.151

[23] Mohamed SS, Awady MEE, Abdelhamid SA, Hamed AA, Salama AAA, Selim MS. Study of exopolysaccharide produced by *Streptomyces* Rochie strain OF1 and its effect as ameliorative on osteoarthritis in rats via inhibiting TNF-α/COX2 pathway. J Genet Eng Biotechnol. 2023. 10.1186/s43141-023-00471-3. 36757520 10.1186/s43141-023-00471-3 PMC9911575

[24] Dubois M, Gilles K, Hamilton JK, Rebers PA, Smith F. A colorimetric method for the determination of sugars. Nature. 1951;168:167. 10.1038/168167a0. 14875032 10.1038/168167a0

[25] DuBois Michel, Gilles KA, Hamilton JK, Rebers PA, Smith Fred. Colorimetric method for determination of sugars and related substances. Anal Chem. 1956;28:350–6. 10.1021/ac60111a017.

[26] Dodgson KS, Price RG. A note on the determination of the ester sulphate content of sulphated polysaccharides. Biochem J. 1962;84:106–10. 13886865 10.1042/bj0840106 PMC1243628

[27] Filisetti-Cozzi TM, Carpita NC. Measurement of uronic acids without interference from neutral sugars. Anal Biochem. 1991;197:157–62. 10.1016/0003-2697(91)90372-z. 1952059 10.1016/0003-2697(91)90372-z

[28] Randall RC, Phillips GO, Williams PA. Fractionation and characterization of gum from acacia Senegal. Food Hydrocolloids. 1989;3:65–75. 10.1016/S0268-005X(89)80034-7.

[29] Ray B. Polysaccharides from enteromorpha compressa: isolation, purification and structural features. Carbohydr Polym. 2006;66:408–16. 10.1016/j.carbpol.2006.03.027.

[30] Meier J, Theakston RDG. Approximate LD50 determinations of snake venoms using eight to ten experimental animals. Toxicon. 1986;24:395–401. 10.1016/0041-0101(86)90199-6. 3715904 10.1016/0041-0101(86)90199-6

[31] Hamed AA, Kabary H, Khedr M, Emam AN. Antibiofilm, antimicrobial and cytotoxic activity of extracellular green-synthesized silver nanoparticles by two marine-derived actinomycete. RSC Adv. 2020;10(17):10361–7. 10.1039/C9RA11021F. 35498609 10.1039/c9ra11021f PMC9050352

[32] Brand-Williams W, Cuvelier ME, Berset C. Use of a free radical method to evaluate antioxidant activity. LWT. 1995;28:25–30. 10.1016/S0023-6438(95)80008-5.

[33] Miller NJ, Rice-Evans CA. The relative contributions of ascorbic acid and phenolic antioxidants to the total antioxidant activity of orange and apple fruit juices and blackcurrant drink. Food Chem. 1997;60:331–7.

[34] Oyaizu M, Studies on Products of Browning Reaction. Jap J Nut Diet. 1986;44:307–15. 10.5264/eiyogakuzashi.44.307.

[35] Gülçin I, Küfrevioglu OI, Oktay M, Büyükokuroglu ME. Antioxidant, antimicrobial, antiulcer and analgesic activities of nettle (Urtica dioica L). J Ethnopharmacol. 2004;90:205–15. 10.1016/j.jep.2003.09.028. 15013182 10.1016/j.jep.2003.09.028

[36] Lo Scalzo R. EPR free radical scavenging activity on superoxide, hydroxyl and tert–butyl hydroperoxide radicals by common hydrophilic antioxidants: effect of mixing and influence of glucose and citric acid. Eur Food Res Technol. 2021;247:2253–65. 10.1007/s00217-021-03785-z.

[37] Adithya ES, Lakshmi MS, Hephzibah P, Sasikumar JM. In vitro antioxidant, anti-lipid peroxidation activities and HPLC analysis of methanol extracts from bark and stem of mahonia Leschenaultia Takeda. Asian J Plant Sci Res. 2013;3:116–26.

[38] Lee G, Choi TW, Kim C, Nam D, Lee S-G, Jang H-J, et al. Anti-inflammatory activities of *reynoutria elliptica* through suppression of mitogen-activated protein kinases and nuclear factor-κb activation pathways. Immunopharmacol Immunotoxicol. 2012;34(3):454–64. 10.3109/08923973.2011.619195. 21961440 10.3109/08923973.2011.619195

[39] Mosmann T. Rapid colorimetric assay for cellular growth and survival: application to proliferation and cytotoxicity assays. J Immunol Methods. 1983;65:55–63. 10.1016/0022-1759(83)90303-4. 6606682 10.1016/0022-1759(83)90303-4

[40] Piran M, Vakilian,Saeid P, ,Mehran M-S. ,Abdollah, Hosseinzadeh, Simzar, and Ardeshirylajimi A. In vitro fibroblast migration by sustained release of PDGF-BB loaded in Chitosan nanoparticles incorporated in electrospun nanofibers for wound dressing applications. Artif Cells Nanomed Biotechnol. 2018;46:511–20. 10.1080/21691401.2018.1430698. 29361857 10.1080/21691401.2018.1430698

[41] Randazzo W, Falcó I, Aznar R, Sánchez G. Effect of green tea extract on enteric viruses and its application as natural sanitizer. Food Microbiol. 2017;66:150–6. 10.1016/j.fm.2017.04.018. 28576363 10.1016/j.fm.2017.04.018

[42] Vijayan P, Hariharapura R, Godavarthi A, So D, Suresh B. Antiviral activity of medicinal plants of Nilgiris. Indian J Med Res. 2004;120:24–9. 15299228

[43] Cotarelo M, Catalán P, Sánchez-Carrillo C, Menasalvas A, Cercenado E, Tenorio A, et al. Cytopathic effect inhibition assay for determining the in-vitro susceptibility of herpes simplex virus to antiviral agents. J Antimicrob Chemother. 1999;44:705–8. 10.1093/jac/44.5.705. 10552991 10.1093/jac/44.5.705

[44] Kanmani P, Satish kumar R, Yuvaraj N, Paari KA, Pattukumar V, Arul V. Production and purification of a novel exopolysaccharide from lactic acid bacterium *Streptococcus phocae* PI80 and its functional characteristics activity *in vitro*. Bioresour Technol. 2011;102:4827–33. 10.1016/j.biortech.2010.12.118. 21300540 10.1016/j.biortech.2010.12.118

[45] Sun R, Fang JM, Goodwin A, Lawther JM, Bolton AJ. Fractionation and characterization of polysaccharides from Abaca fibre. Carbohydr Polym. 1998;37:351–9. 10.1016/S0144-8617(98)00046-0.

[46] Cheng A, Wan F, Jin Z, Wang J, Xu X. Nitrite oxide and inducible nitric oxide synthase were regulated by polysaccharides isolated from *glycyrrhiza uralensis* fisch. J Ethnopharmacol. 2008;118:59–64. 10.1016/j.jep.2008.03.002. 18434050 10.1016/j.jep.2008.03.002

[47] Meyers MA, Chen P-Y, Lin AY-M, Seki Y. Biological materials: structure and mechanical properties. Prog Mater Sci. 2008;53:1–206. 10.1016/j.pmatsci.2007.05.002. 10.1016/j.jmbbm.2008.02.003 19627786

[48] Wang C, Chen Y, Hu M, Ding J, Xu C, Wang R. In vitro antioxidant activities of the polysaccharides from tricholoma lobayense. Int J Biol Macromol. 2012;50:534–9. 10.1016/j.ijbiomac.2012.01.005. 22305884 10.1016/j.ijbiomac.2012.01.005

[49] Guo Z, Xing R, Liu S, Yu H, Wang P, Li C, et al. The synthesis and antioxidant activity of the schiff bases of chitosan and carboxymethyl chitosan. Bioorg Med Chem Lett. 2005;15:4600–3. 10.1016/j.bmcl.2005.06.095. 16122924 10.1016/j.bmcl.2005.06.095

[50] Milenković D, Dimić D, Avdović E, Simijonović D, Vojinović R, Marković Z. A thermodynamic and kinetic HO radical scavenging study and protein binding of Baicalein. J Chem Thermodyn. 2023;185:107110. 10.1016/j.jct.2023.107110.

[51] Khalil MA, Sonbol FI, Al-Madboly LA, Aboshady TA, Alqurashi AS, Ali SS. Exploring the therapeutic potentials of exopolysaccharides derived from lactic acid bacteria and bifidobacteria: antioxidant, antitumor, and periodontal regeneration. Front Microbiol. 2022;13:803688. 10.3389/fmicb.2022.803688. 35547125 10.3389/fmicb.2022.803688 PMC9082500

[52] Ghanaim AM, Mohamed HI, El–Ansary AE. Production and characterization of exopolysaccharides from *Pseudomonas aeruginosa* AG01 with some medical potential applications. Microb Cell Fact. 2025;24:107. 10.1186/s12934-025-02730-z. 40369557 10.1186/s12934-025-02730-z PMC12077034

[53] Hazra P, Ghosh S, Roy P, Sinha D, Kesh GS. Antioxidant activity of exopolysaccharides from marine cyanobacteria. Microbe. 2025;8:100430. 10.1016/j.microb.2025.100430.

[54] Cirino G. Multiple controls in inflammation. Extracellular and intracellular phospholipase A2, inducible and constitutive cyclooxygenase, and inducible nitric oxide synthase. Biochem Pharmacol. 1998;55:105–11. 10.1016/s0006-2952(97)00215-3. 9448732 10.1016/s0006-2952(97)00215-3

[55] Mahmoud YA-G, El-Naggar ME, Abdel-Megeed A, El-Newehy M. Recent advancements in microbial polysaccharides: synthesis and applications. Polym (Basel). 2021;13:4136. 10.3390/polym13234136. 10.3390/polym13234136 PMC8659985 34883639

[56] Yermagambetova A, Tazhibayeva S, Takhistov P, Tyussyupova B, Tapia-Hernández JA, Musabekov K. Microbial polysaccharides as functional components of packaging and drug delivery applications. Polymers. 2024;16:2854. 10.3390/polym16202854. 39458682 10.3390/polym16202854 PMC11511474

[57] Moscovici M. Present and future medical applications of microbial exopolysaccharides. Front Microbiol. 2015;6:1012. 10.3389/fmicb.2015.01012. 26483763 10.3389/fmicb.2015.01012 PMC4586455

[58] Emam AA, Abo-Elkhair SM, Sobh M, El-Sokkary AMA. Role of exopolysaccharides (EPSs) as anti-Mir-155 in cancer cells. Heliyon. 2021;7:e06698. 10.1016/j.heliyon.2021.e06698. 33869874 10.1016/j.heliyon.2021.e06698 PMC8045046

[59] Amjad E, Sokouti B, Asnaashari A. A systematic review of anti-cancer roles and mechanisms of kaempferol as a natural compound. Cancer Cell Int. 2022;22:260. 10.1186/s12935-022-02673-0. 35986346 10.1186/s12935-022-02673-0 PMC9392350

[60] Ramamurthy V, Vallinachiyar C. Apoptosis of human breast cancer cells (MCF-7) induced by polysacccharides produced by bacteria. J Cancer Sci Therapy. 2013;5:031–4. 10.4172/1948-5956.1000181.

[61] Selim MS, Mohamed SS, Asker MM, Salama AAA, Abdallah HMI, Yassen NN. Production and characterization of exopolysaccharide from marine Bacillus sp. MSHN2016 with studying its effect on isoniazid/ rifampicin-induced hepatic and renal toxicities in rats. J App Pharm Sci. 2018;8,:001–11. 10.7324/JAPS.2018.8801.

[62] Mohamed S, Amer S, Selim M, Rifaat H. Characterization and applications of exopolysaccharide produced by marine *Bacillus altitudinis* MSH2014 from Ras Mohamed, Sinai, Egypt. Egypt J Basic Appl Sci. 2018. 10.1016/j.ejbas.2018.05.009.

[63] Tukenmez U, Aktas B, Aslim B, Yavuz S. The relationship between the structural characteristics of lactobacilli-EPS and its ability to induce apoptosis in colon cancer cells in vitro. Sci Rep. 2019;9:8268. 10.1038/s41598-019-44753-8. 31164685 10.1038/s41598-019-44753-8 PMC6547643

[64] Chipuk JE, Maurer U, Green DR, Schuler M. Pharmacologic activation of p53 elicits Bax-dependent apoptosis in the absence of transcription. Cancer Cell. 2003;4:371–81. 10.1016/s1535-6108(03)00272-1. 14667504 10.1016/s1535-6108(03)00272-1

[65] El-Deeb NM, Yassin AM, Al-Madboly LA, El-Hawiet A. A novel purified *Lactobacillus acidophilus* 20079 exopolysaccharide, LA-EPS-20079, molecularly regulates both apoptotic and NF-κB inflammatory pathways in human colon cancer. Microb Cell Fact. 2018;17:29. 10.1186/s12934-018-0877-z. 29466981 10.1186/s12934-018-0877-z PMC5820793

[66] Liu G, Kuang S, Wu S, Jin W, Sun C. A novel polysaccharide from *sargassum integerrimum* induces apoptosis in A549 cells and prevents angiogensis *in vitro* and *in vivo*. Sci Rep. 2016;6:26722. 10.1038/srep26722. 27216943 10.1038/srep26722 PMC4877640

[67] Ogura F, Hayashi K, Lee J-B, Kanekiyo K, Hayashi T. Evaluation of an edible blue-green alga, *aphanothece sacrum*, for its inhibitory effect on replication of herpes simplex virus type 2 and influenza virus type A. Biosci Biotechnol Biochem. 2010;74:1687–90. 10.1271/bbb.100336. 20699552 10.1271/bbb.100336

[68] Saad MH, Sidkey NM, El-Fakharany EM. Characterization and optimization of exopolysaccharide extracted from a newly isolated halotolerant cyanobacterium, *acaryochloris* Al-azhar MNE ON864448.1 with antiviral activity. Microb Cell Fact. 2024;23:117. 10.1186/s12934-024-02383-4. 38644470 10.1186/s12934-024-02383-4 PMC11034128

[69] Cimino P, Bifulco G, Casapullo A, Bruno I, Gomez-Paloma L, Riccio R. Isolation and NMR characterization of rosacelose, a novel sulfated polysaccharide from the sponge mixylla rosacea. Carbohydr Res. 2001;334:39–47. 10.1016/s0008-6215(01)00141-0. 11470249 10.1016/s0008-6215(01)00141-0

[70] Muschin T, Budragchaa D, Kanamoto T, Nakashima H, Ichiyama K, Yamamoto N, et al. Chemically sulfated natural galactomannans with specific antiviral and anticoagulant activities. Int J Biol Macromol. 2016;89:415–20. 10.1016/j.ijbiomac.2016.05.005. 27154517 10.1016/j.ijbiomac.2016.05.005

