# Supplementary material for: Revealing the therapeutic potential of bioactive exopolysaccharide (EPSS10) derived from Streptomyces sp. MAE
Source: BMC Microbiol. 2025 Nov 15;25:749. doi: 10.1186/s12866-025-04462-x (PMC13235160; doi:10.1186/s12866-025-04462-x)
Supplement: Supplementary file 1 — Supplementary Material 1. [file 12866_2025_4462_MOESM1_ESM.pdf]

Revealing the medicinal potential of bioactive exopolysaccharide (EPSS10) produced from *Streptomyces* sp. MAE

Esraa S. Refat[1‡], Attia A. Attia[1‡], Mohamed E. El Awady[2*], Mohamed Ali[3], Ahmed A. Hamed[4], Mohamed A. Nasr-Eldin[1*]

[1] Botany and Microbiology Department, Faculty of Science, Benha University, Benha 13511, Egypt.

[2] Microbial Biotechnology Department, National Research Centre, El-Buhouth St. 33, Dokki-Cairo 12622, Egypt.

[3] Biochemistry Department, Faculty of Science, Zagazig University, Zagazig, Egypt.

[4] Microbial Chemistry Department, National Research Centre, El-Buhouth St. 33, Dokki-Cairo 12622, Egypt.

‡Both authors are equally contributed in the first

***Corresponding authors: Mohamed E. El Awady& Mohamed A. Nasr-Eldin**

E-mail: Mohamed_elawady82@yahoo.com& mohamed.nasreldin@fsc.bu.edu.eg

ORCID iD: Mohamed E. El Awady https://orcid.org/0000-0001-5155-3949

ORCID iD: Mohamed A. Nasr-Eldin, https://orcid.org/0000-0002-8537-9392

Supplementary Table (1): Primers used for real-time PCR analysis

**No**	**Primer Name**	**Forward primer sequence (5′→3′)**	**Reverse primer sequence (5′→3′)**
1	Bax	5'-TCAGGATGCGTCCACCAAGAAG -3'	5'-TGTGTCCACGGCGGCAATCATC -3'
2	bcl2	5'-ATCGCCCTGTGGATGACTGAGT-3'	5'-GCCAGGAGAAATCAAACAGAGGC-3'
3	Casp9	5'- GTTTGAGGACCTTCGACCAGCT-3'	5'- CAACGTACCAGGAGCCACTCTT -3'
4	p53	5'- CCTCAGCATCTTATCCGAGTGG-3'	5'-TGGATGGTGGTACAGTCAGAGC -3'
5	CYC	5'- AAGGGAGGCAAGCACAAGACTG -3'	5'- CTCCATCAGTGTATCCTCTCCC -3'
6	GAPDH	5'- GTCTCCTCTGACTTCAACAGCG-3'	5'- ACCACCCTGTTGCTGTAGCCAA-3

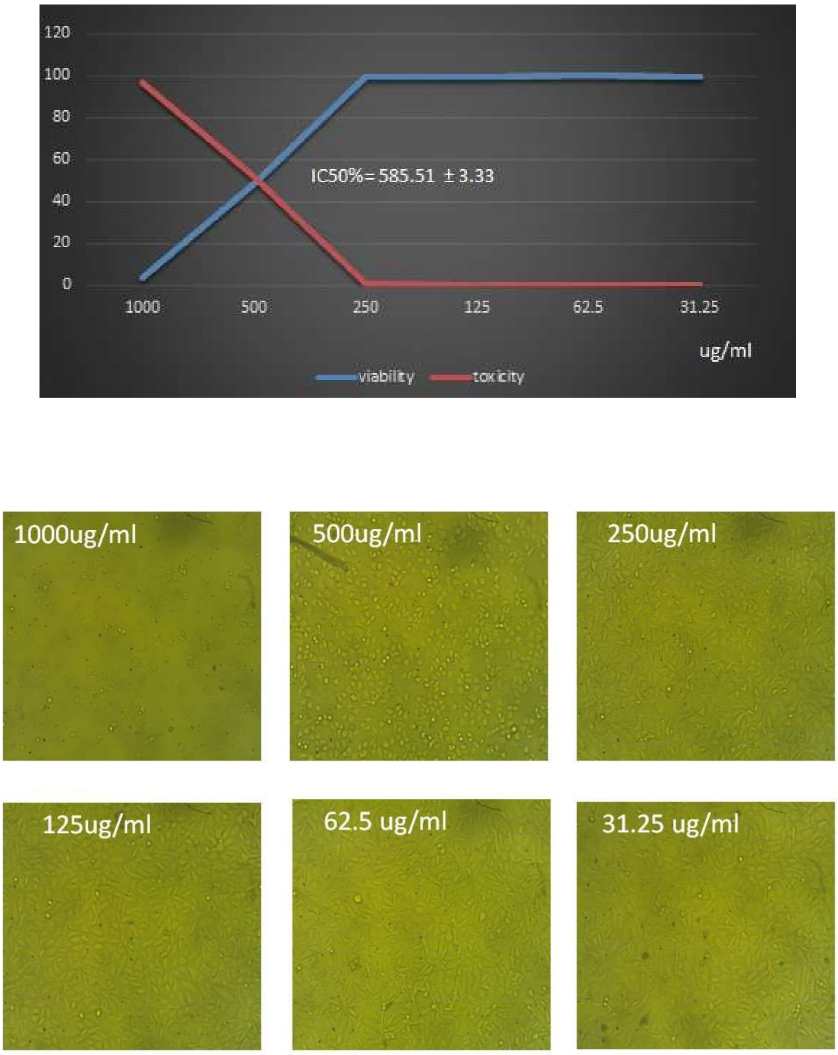

Supplementary Figure (1). Effect of EPSS10 on HepG2 cells at different concentrations

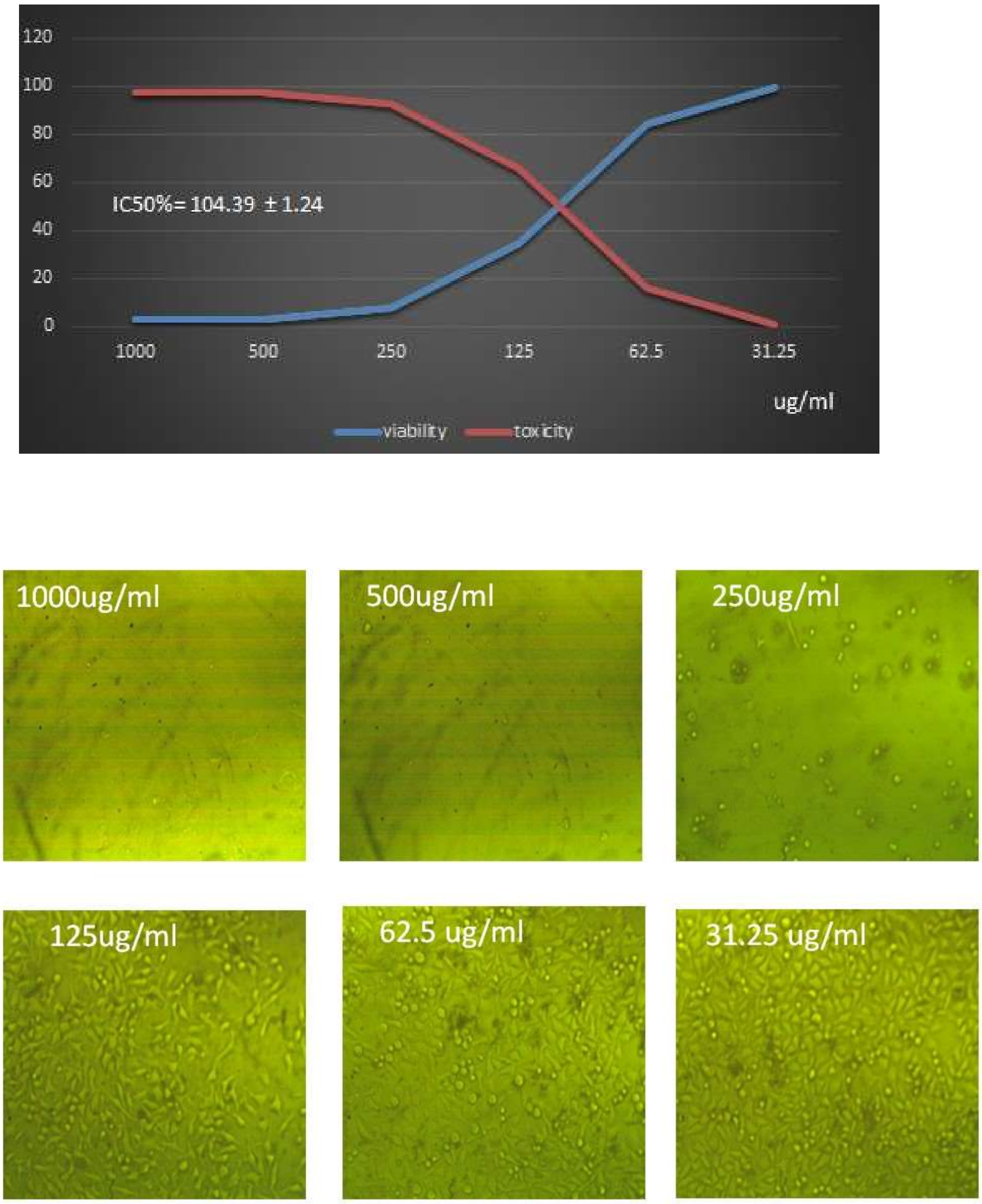

Supplementary Figure (2). Effect of EPSS10 on CaCo2 cells at different concentrations

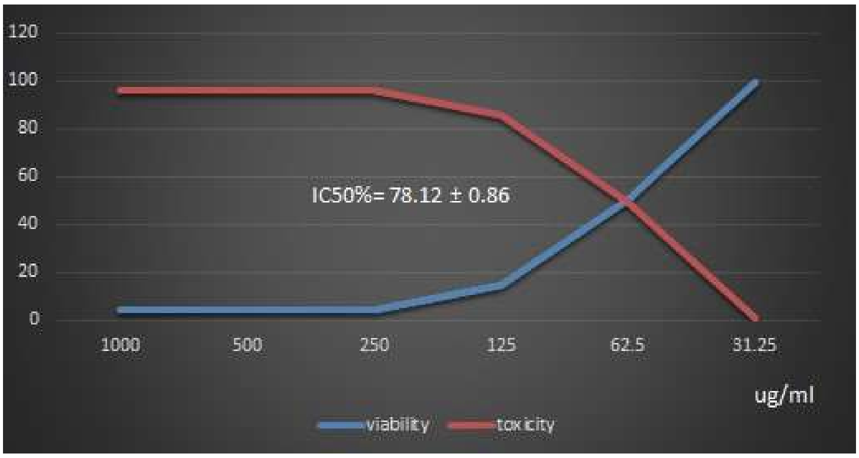

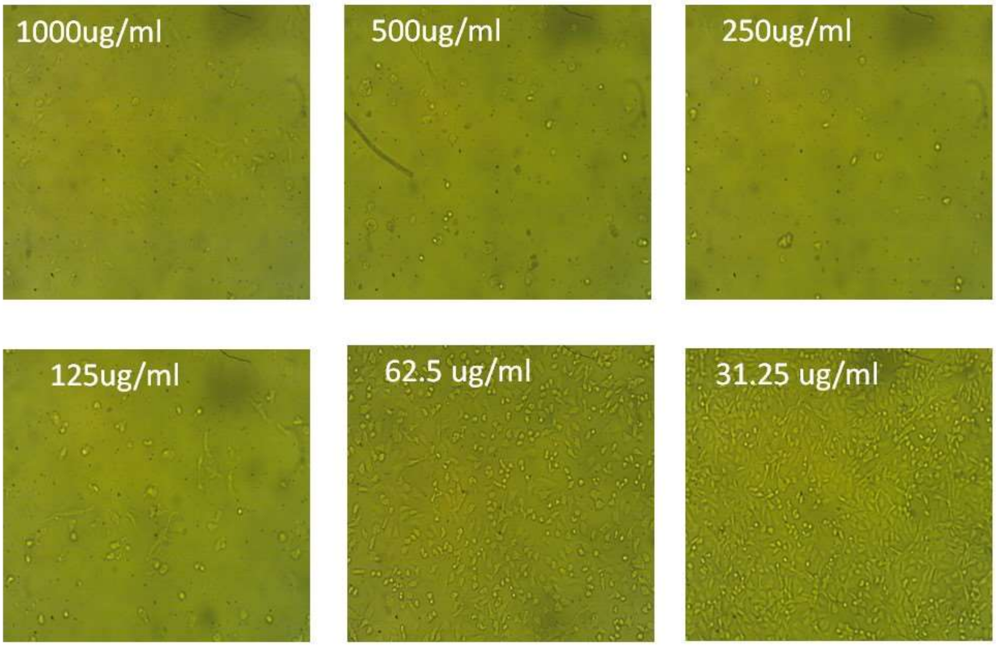

Supplementary Figure (3). Effect of EPSS10 on MCF7 cells at different concentrations

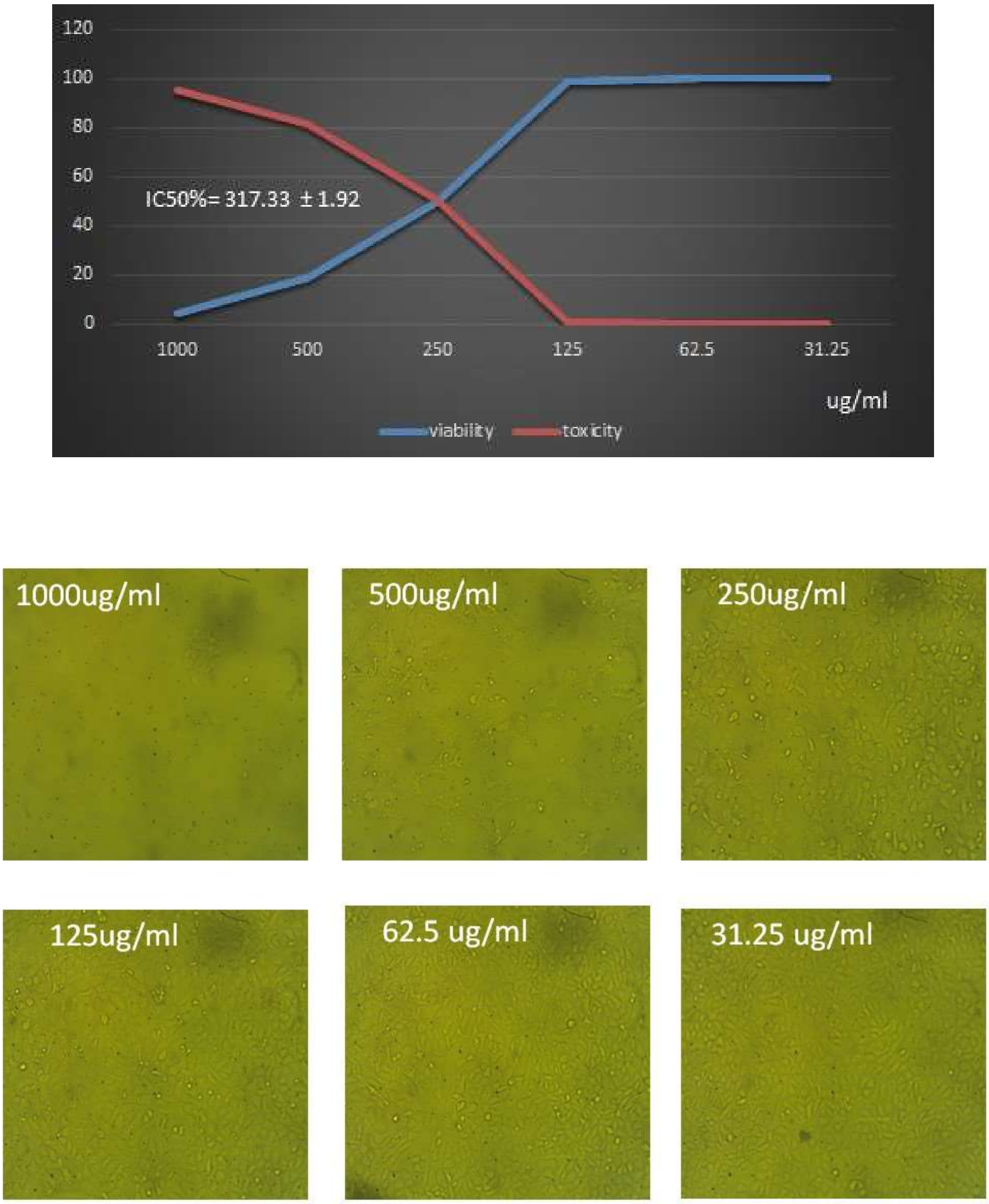

Supplementary Figure (4). Effect of EPSS10 on PC3 cells at different concentrations

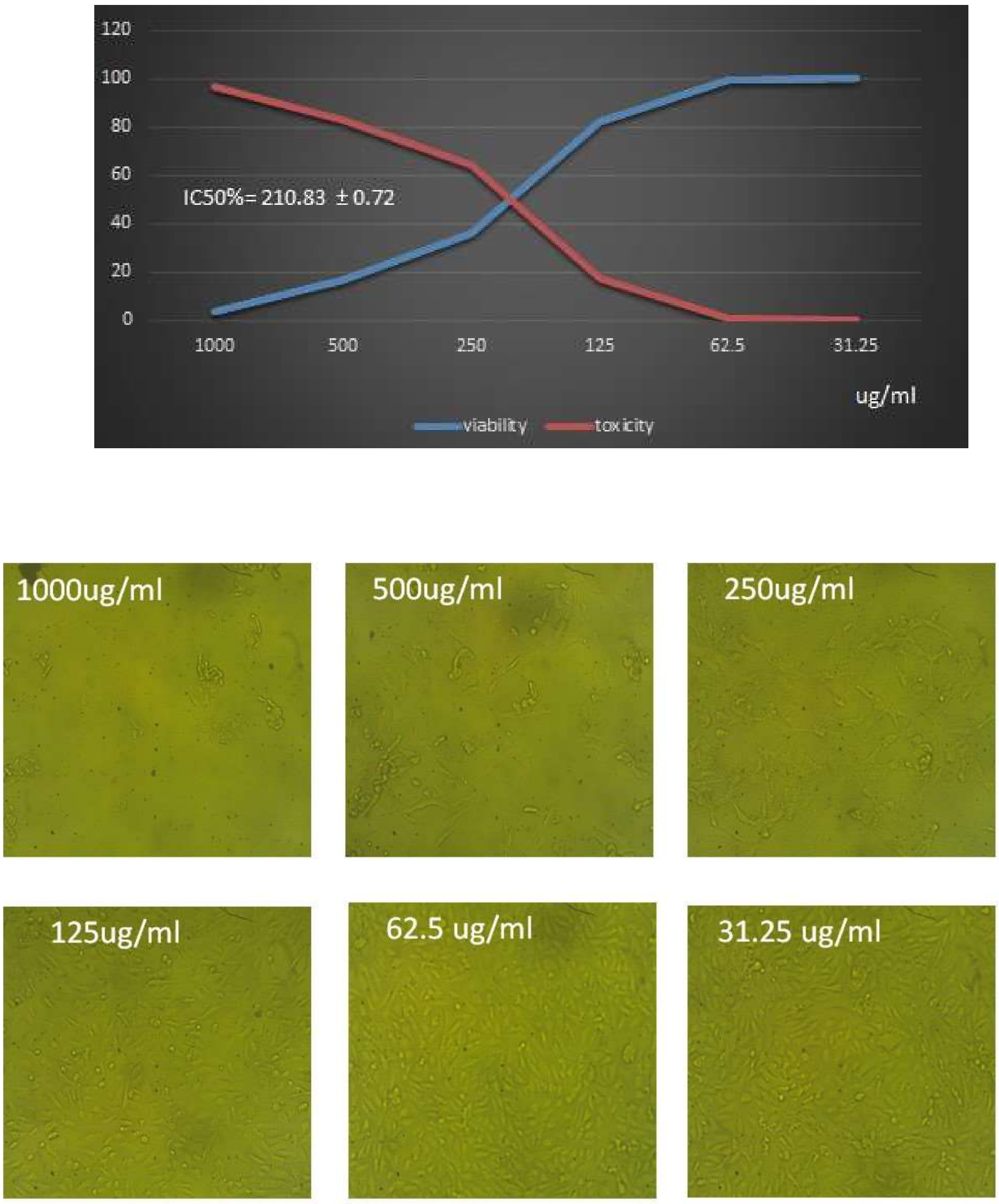

Supplementary Figure (5). Effect of EPSS10 on A-549 cells at different concentrations

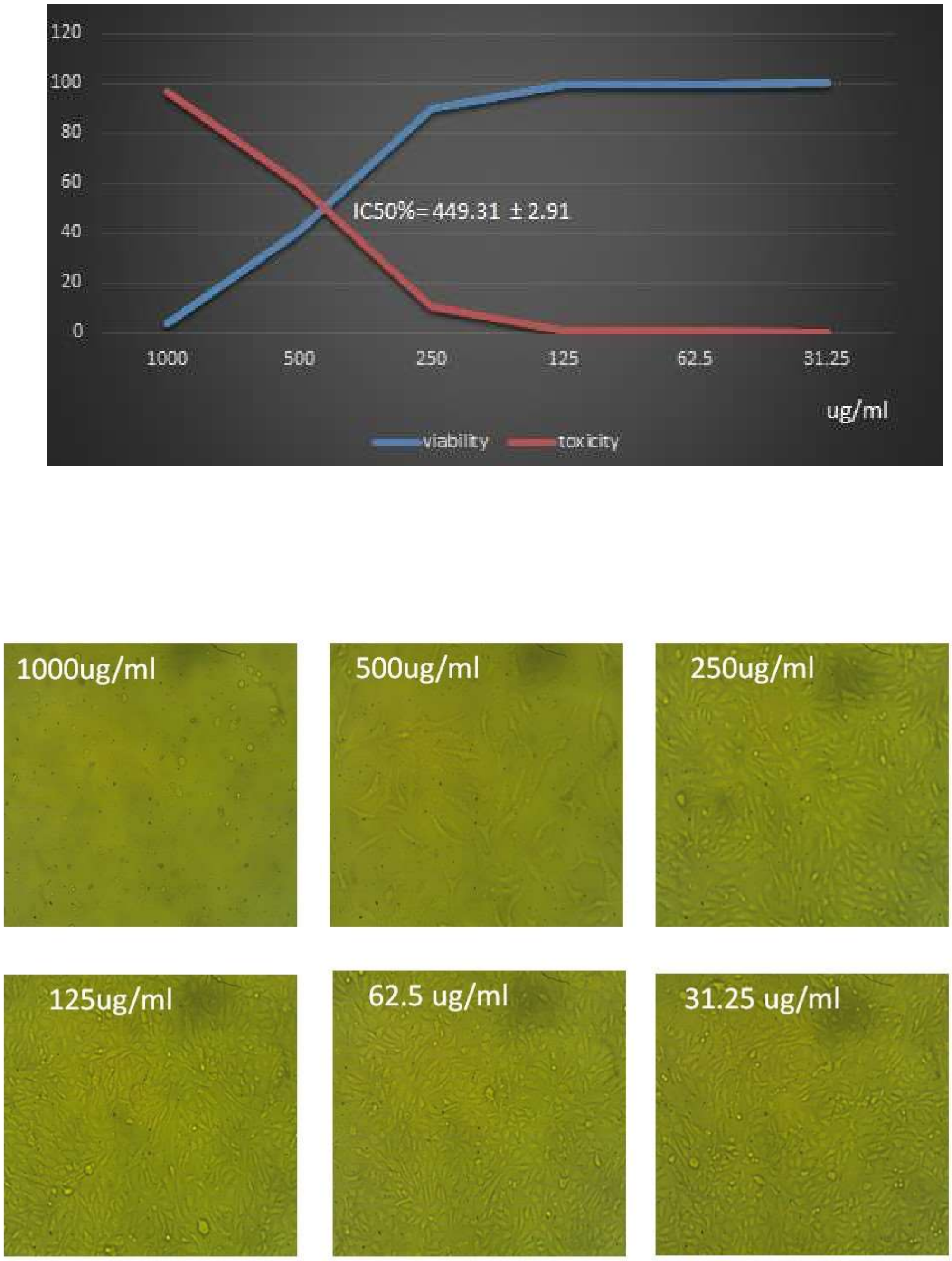

Supplementary Figure (6). Effect of EPSS10 on PANC-1 cells at different concentrations
